# A genetic tradeoff for tolerance to moderate and severe heat stress in US hybrid maize

**DOI:** 10.1371/journal.pgen.1010799

**Published:** 2023-07-06

**Authors:** Aaron Kusmec, Lakshmi Attigala, Xiongtao Dai, Srikant Srinivasan, Cheng-Ting “Eddy” Yeh, Patrick S. Schnable

**Affiliations:** 1 Department of Agronomy, Iowa State University; Ames, Iowa, United States of America; 2 Department of Statistics, Iowa State University; Ames, Iowa, United States of America; 3 Plant Sciences Institute, Iowa State University; Ames, Iowa, United States of America; Gregor Mendel Institut für Molekulare Pflanzenbiologie GmbH: Gregor Mendel Institut fur Molekulare Pflanzenbiologie GmbH, AUSTRIA

## Abstract

Global climate change is increasing both average temperatures and the frequencies of extreme high temperatures. Past studies have documented a strong negative effect of exposures to temperatures >30°C on hybrid maize yields. However, these studies could not disentangle genetic adaptation via artificial selection from changes in agronomic practices. Because most of the earliest maize hybrids are no longer available, side-by-side comparisons with modern hybrids under current field conditions are generally impossible. Here, we report on the collection and curation of 81 years of public yield trial records covering 4,730 maize hybrids, which enabled us to model genetic variation for temperature responses among maize hybrids. We show that selection may have indirectly and inconsistently contributed to the genetic adaptation of maize to moderate heat stress over this time period while preserving genetic variance for continued adaptation. However, our results reveal the existence of a genetic tradeoff for tolerance to moderate and severe heat stress, leading to a decrease in tolerance to severe heat stress over the same time period. Both trends are particularly conspicuous since the mid-1970s. Such a tradeoff poses challenges to the continued adaptation of maize to warming climates due to a projected increase in the frequency of extreme heat events. Nevertheless, given recent advances in phenomics, enviromics, and physiological modeling, our results offer a degree of optimism for the capacity of plant breeders to adapt maize to warming climates, assuming appropriate levels of R&D investment.

## Author summary

As climate change increases average temperatures, the heat tolerance of major crops becomes more important to global food security. Past studies in maize have documented strongly negative effects of heat stress on maize yields but without adequate characterization of changes to heat tolerance over time. In this study, we explored changes in maize heat tolerance over the past 80 years of hybrid maize breeding using data from public yield trials. We showed that tolerance to moderate heat stress has increased, but that tolerance to severe heat stress has decreased. Because climate change is expected to increase the incidence of severe heat stress in particular, these findings indicate the need for more detailed genetic and physiological studies of heat tolerance and their incorporation into plant breeding efforts. They also highlight the past successes of plant breeders at adapting maize to moderate heat stress. Overall, our findings suggest directions for further research to mitigate the negative effects of climate change and a tempered optimism for their application.

## Introduction

US maize yields increased substantially over the course of the 20th century [1] due to a combination of genetic improvement and improved agronomic practices that increased tolerance to higher planting densities [2]. Like other crops, maize grows best within specific temperature ranges. Exposure to excessive heat or cold alters normal physiological functions, leading to yield losses. Because global climate change increased and continues to increase the frequency and duration of extreme weather episodes [3–5], growth in global agricultural productivity has slowed since 1961 relative to a counterfactual model lacking climate change [6]. Furthermore, absent sufficient genetic adaptation to extreme temperatures and corresponding changes to agronomics, yield losses are expected to increase [7], leading to the possibility of synchronized shocks to global production systems [8].

Increases in global mean temperatures and alterations to precipitation patterns are expected to increase the incidence of two key, often co-occurring abiotic stresses: heat and drought. Together with soil properties, temperature and precipitation interact to determine water supply and demand dynamics throughout the growing season. Previous analyses have identified a strong, negative, non-linear effect of exposure to temperatures >29–30°C on maize [9–12]. Simulations using process-based crop growth models determined that temperature has a stronger effect on maize yields than precipitation [13] because higher temperatures drive increased plant and atmospheric water demand, overwhelming the positive or negative effects of altered precipitation on soil water availability. Subsequent work has demonstrated the importance of interactions between vapor pressure deficit (driven by temperature and humidity) and root-zone soil moisture for predicting maize yield anomalies [12,14,15].

Much attention has recently been paid to the adaptive potential of shifting planting dates to maintain historical crop cycle durations [16,17] coupled with irrigation and/or regional transfers of cultivars [18,19]. The goal of such a strategy is to leverage phenological variability to reduce exposure to heat and drought stress during critical developmental periods for maize, namely, anthesis and grain-fill. However, such a strategy would depend on the ability of plant breeders to leverage genetic variability for flowering time [explicitly recognized by [17]], which has a non-linear dependence on temperature [20]. Yield, as a measure of organismal fitness, is integrative [21], dependent on the contributions of multiple interacting biotic and abiotic factors that influence crop cycle duration in addition to other important, intermediate traits. While crop cycle duration adaptation will undoubtedly be a component of any future adaptative strategy, it cannot by itself insulate yields from the effects of higher average temperatures and the increased probability of extreme heat events. Such an adaptation is an example of an avoidance strategy whereby the stress is not encountered or reduced but does not necessarily address tolerance strategies whereby the plant endures encountered stress by reducing deleterious effects. Thus, improvements in the heat tolerance of maize hybrids should also be a target of future adaptive strategies.

The possibility of realizing such adaptations depends on the presence of genetic variation for heat tolerance, which is a necessary condition for genetic gain in any trait, and the structure of the genetic covariances between different components of fitness. Concerns regarding the maintenance and exploitation of genetic variation have increased in the last two decades following the widespread adoption of genomic selection [22], which—depending on the mate-pairing schemes employed—alters the dynamics of genetic (co-)variances relative to phenotypic selection [23–26]. This is to say that the multivariate genetic architecture of present maize populations has been shaped by historical selection; it is from this base that breeding decisions will be made to adapt maize to a warming climate; and the dynamics of genetic (co-)variances have changed in the recent past. As such, it is important to understand the adaptive (or, possibly, maladaptive) trajectory of hybrid maize as a means for understanding the constraints within which we work at the present day.

Despite annual increases in maize yields for the past 90 years, two studies suggest that concerns about the trajectory of hybrid maize heat tolerance are more than theoretical. The first study [27], using 100 years of yield reports for the US state of Indiana, documented temporal variation in the effect of cumulative degree days >29°C on maize yields with a peak near 1960, which coincides with the adoption of single-cross hybrids, followed by increasing susceptibility to heat stress. The second study [28] updated the models and data of [9] to predict maize yield anomalies in the eastern US during the 2012 heat wave using temperature effects estimated from 60 years of data. While one might expect that increases in maize yields have been partly due to increases in heat tolerance and that the models of [28] would therefore over-predict yield losses in 2012, they in fact *under*-predict actual yield losses, suggesting that modern maize hybrids are potentially *less* heat tolerant than historical varieties. However, both of these studies aggregate exposure to all temperatures >29°C, assuming that trends in heat tolerance are identical across a range of heat stress severities. Furthermore, the data used by these studies do not allow them to address the question of maladaptation of hybrid maize to high temperatures by separating the effects of genetics and agronomic practices.

The studies cited above characterize the impacts of exposure to various temperatures on yields by combining national agricultural statistics (such as county-level yields reported by the United States Department of Agriculture, National Agricultural Statistics Service [USDA-NASS]) with econometric panel-data models [29,30]. Importantly, these aggregated data do not include information on the specific hybrids grown by individual farmers within a county, which are expected to be heterogeneous, and studies reliant on these data cannot, therefore, directly incorporate genetic variation into estimates of temperature responses as noted above. While the panel-data approach can capture the effects of *global* (i.e., genetic and agronomic) adaptation over time [31] [see also the long-differences approach of [32]], these studies are unable to specifically address the existence and nature of genetic adaptation. Attempts to estimate broad adaptive trends through temporal subsets [9] or weather-time interaction terms [12] typically fail to find statistically significant evidence for such trends. This failure to identify adaptive trends is probably due to the genetic heterogeneity of the observational units (typically counties) as studies that do incorporate explicit genetic information in wheat find evidence for genetic trends [33,34]. Stated differently, summary data measure the response at the level of the entire county without accounting for the effects of hybrids, which are treatments applied at the level of individual farmers’ fields, inappropriately conflating the observational and experimental units.

Despite this, the panel data framework has several advantages that address important issues related to the control of spatial and temporal variation [35,36] that can impact the estimation of selection responses. Through the incorporation of location effects, this framework captures time-invariant but spatially variable unobserved effects such as soil quality. Through the incorporation of time effects (possibly stratified by larger geographical regions), this framework captures spatially invariant but temporally variable unobserved effects such as fertilizer usage or planting density. Inclusion of both dimensions (a two-way panel data model as opposed to cross-sectional [location only] or time-series [time only] analyses) is an effective means to reduce omitted variables bias and increase degrees of freedom for statistical analyses [30]. In the context of quantitative genetics, these are classified as common environmental effects and separated from genetic effects by the inclusion of additional random effects terms in the animal model [37].

Studies in wheat [33,34] and sorghum [38] have combined the panel-data approach with on-farm and variety testing data that includes information on cultivars to estimate cultivar-specific heat responses in these two crops, demonstrating either a trend toward increasing heat sensitivity (wheat) or no trend in heat sensitivity (sorghum). In maize, cross-sectional studies have identified recent increases in weather (particularly water availability) sensitivity [12,14] and long-term, complex trends in heat sensitivity, which has been increasing since 1960 [27], using aggregated data. The advantage of the wheat and sorghum studies compared to earlier panel data models is that the observational units are now identical with the experimental units: individual cultivars grown in experimental plots rather than genetically heterogeneous, geographical units. This permits the estimation of cultivar-specific coefficients on the weather variables that are conditioned on the time-(in)variant effects.

However, these studies suffer from three limitations. First, the referenced studies rarely use data collected before 1985 [[27] is the sole exception] due to data availability [14,33,38] and/or the use of modern remote sensing technologies [12]. Thus, they fail to capture climate trends differing from those of the recent past [39], including the first half of the 20th century before climate change is estimated to have begun affecting agricultural productivity [6]. Second, the referenced studies use linear or piece-wise linear specifications for temperature response functions [with the exception of [14]]. Although some of these are more complex, admitting variable effects of heat at different periods of the growing season [33,34,38], they are still simplifications of complex physiological responses. The parametric assumptions imposed by these studies limit the flexibility and nature of potential non-linearities in the estimated temperature response functions, which can lead to misleading conclusions regarding genetic variation for such traits [40] and obscure non-intuitive responses to selection due to the existence of non-linear constraints [41,42]. Although [28] uses a cubic B-spline basis and temporally-variable temperature responses, their use of summary data does not allow the estimation of genetic trends. Third, the genetic variation introduced into estimates of the temperature response function in [33,34,38] is limited to responses to the most extreme temperatures. This provides a limited view of the genetic variation in temperature responses within the studied populations and limits inferences on selection for responses to all temperatures experienced during the growing season. Additionally, this can bias projections of future yield losses due to climate change by not allowing for the possibility that cultivars are differentially responsive to optimum temperatures.

As done previously for wheat [33,34] and sorghum [38], we combine the two-way panel-data approach with records of crop variety testing programs operated by US land-grant universities. These programs began, for hybrid maize, in the 1930s and continue to the present day. They compare the yields of (pre-)commercial and public maize hybrids across multiple locations within individual US states to help farmers make informed decisions about which hybrids to plant based on performance over a short sample of the local climate. Records for these trials are publicly available; encompass both publicly and privately developed maize hybrids; include information on single-year yields for individual hybrids at each trial location; and cover multiple regimes of climate change across the 20th century.

Here, we report on the collection and curation of public yield trial data and an associated climate dataset. The combined dataset covers 81 years of trials over four US states and includes 4,730 maize hybrids. In contrast to prior studies, we specify a functional linear mixed effects model using a B-spline basis that decreases constraints on the shape of the response and allows us to model hybrid-specific temperature response functions, capturing the genetic variance-covariance structure of responses across observed temperatures. This allows us to: (1) test for the effects of selection on temperature response functions over 81 years of hybrid maize breeding, and (2) investigate the modes of genetic variation in temperature responses.

## Results

### Historical data overview

**Fig 1:**
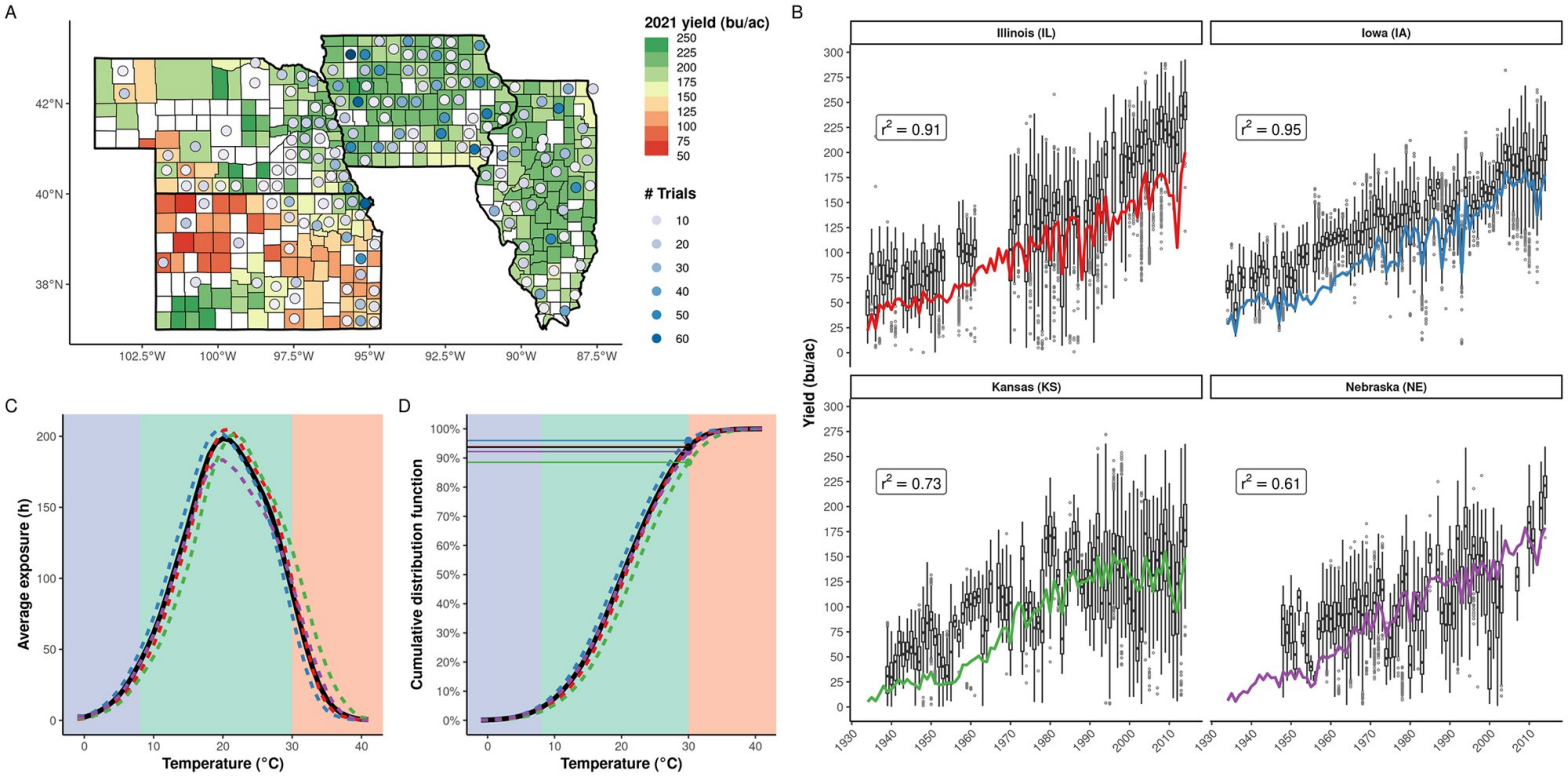
Characterization of the combined yield trial and climate datasets. **(A)** Geographical distribution of trials for 1934–2014. Counties are colored by 2021 average maize yields (bu/a) reported by USDA-NASS. White indicates no data. Trials are indicated by points, and the color indicates the number of trials occurring in that county (maximum 80 trials). Some trials fall within the bounds of an adjacent state but are not administered by that state. The map was generated using shapefiles distributed with the ‘maps’ R package (https://cran.r-project.org/package=maps). **(B)** Distribution of yields from all trials in each year stratified by state. Boxplots indicate the first and third quartiles and median yield in each year, and whiskers indicate 1.5 times the interquartile range. Solid lines indicate the average state yield for each year as reported by USDA-NASS. Pearson’s correlation (*r*^2^) between the average trial yield and state average yield within each state is reported in the insets. **(C)** Average exposure time for the growing season across all years and trials in 1°C temperature bins. The solid line indicates the average across all 2,581 trials, and dashed lines indicate the average for each state [colors correspond to panel **(B)**]. The background indicates exposure to temperatures below (cold stress), in, and above (heat stress) the optimal range for maize as typically defined for growing degree days (GDD, 8–30°C). **(D)** Cumulative distribution functions of average exposure time for the growing season as described in panel **(C)**. Dots and horizontal lines indicate the proportion of the average growing season for which hybrids experience heat stress (>30°C).

We collected trial results from four US states for 81 years (1934–2014; Fig 1A). Our dataset contains 172 unique trial locations comprising a total of 2,581 non-irrigated environments (location-year combinations); 4,730 hybrids; and 175,805 total observations. Average trial yields are generally higher than and moderately to highly correlated with the corresponding state averages (Fig 1B). Mean hybrid yields and standard deviations have increased over time within all states with the rate of increase of the former being greater, leading to a reduction in the coefficient of variation of yields over time in Illinois and Iowa and no trend in Kansas and Nebraska (S3 Fig).

Trials were linked with historical weather records to derive the distribution of time exposed to temperatures in 1°C bins following [9,43] (S1 Fig). The average exposure distribution is slightly left-skewed with the majority of the growing season exposing maize to temperatures within the typical optimal range—8–30°C—as defined by GDD (Fig 1C; green region). During 1934–2014, these trials were exposed to 218±3.1 h/yr (mean ± s.e.; 6.38±0.09% of the growing season) of temperatures >30°C. Exposure to extreme temperatures varies by state (Fig 1C and 1D) and county (S4 Fig) with less exposure to heat stress in northern latitudes. Nevertheless, exposure to heat stress is well distributed across the various counties, years, and hybrids in our dataset with a minimum of 6,355 observations for the most extreme temperatures (>41°C) and at least 10,000 observations for all other temperatures (S5 Fig).

### Resistance to severe heat stress has declined

We modeled the temperature responses of the 4,730 hybrids using a functional linear mixed effects regression model [44,45] where the temperature response function was composed of a fixed population mean function and random functions for each hybrid [see “Methods,” Eq (1); S2 Fig]. To reduce assumptions about the form of the temperature response functions, we used a cubic B-spline basis with three internal knots (S6 Fig) to model the temperature exposure distributions and temperature response functions [46] [see “Methods” for a description of an alternative parameterization using constant B-splines, equivalent to the step model of, e.g., [9,11]]. Potentially confounding factors for the estimation of temperature response functions are included as covariates in the model, including fixed effects of Julian planting date (S7 Fig) and season-total precipitation (S8 Fig); random effects of county to capture location-specific, time-invariant effects such as soil type (S9 Fig); and random regressions on year stratified by state to capture changes in agronomic practices (S10 Fig). We used block bootstrapping on years to control for spatial correlation within years and estimate 95% confidence bands (CBs) on the coefficient functions.

**Fig 2:**
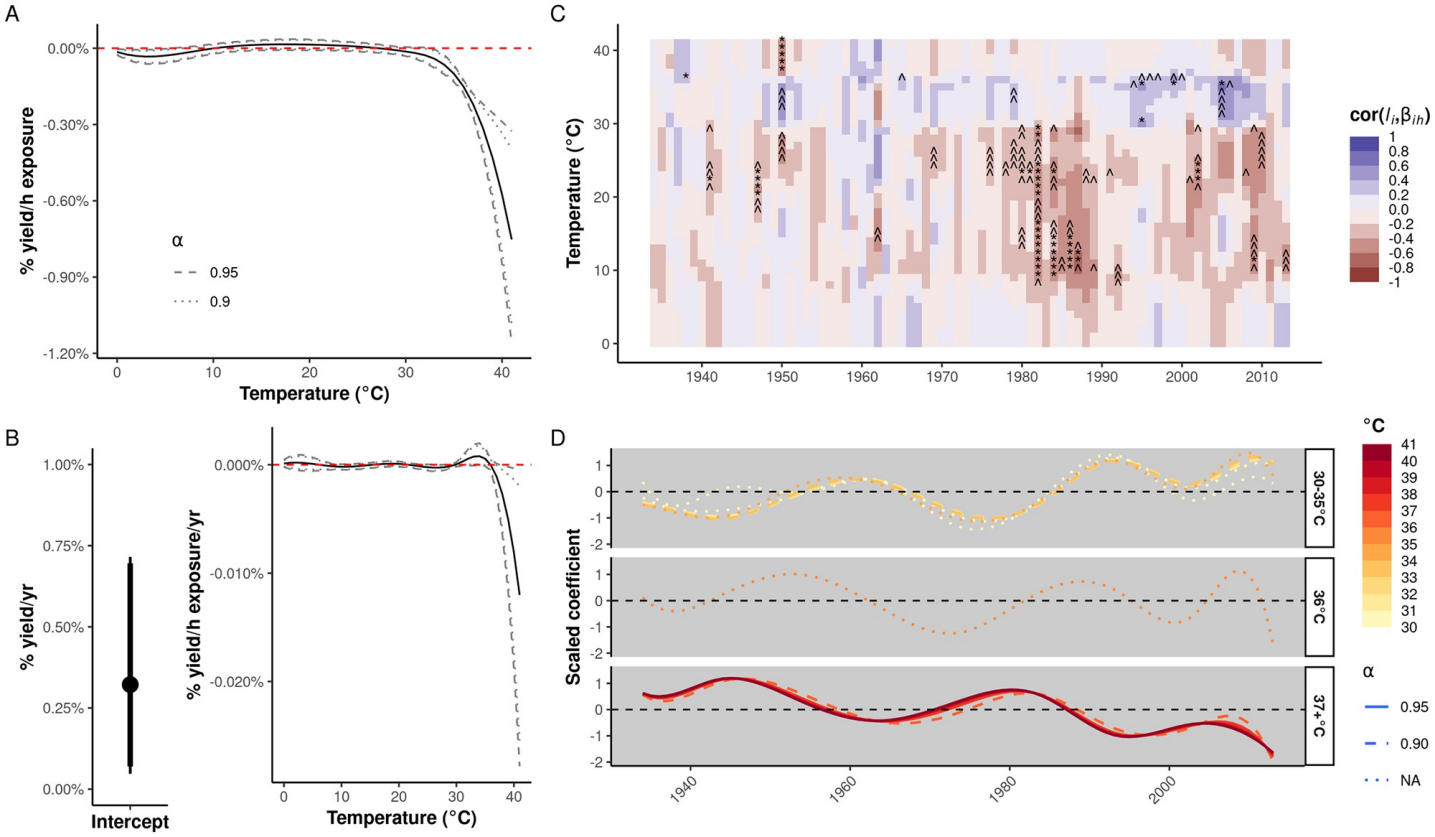
Quantification of directional selection on temperature response functions. Results shown in this figure are for exposure distributions and response functions parameterized as cubic B-splines (see “Methods” and S11 Fig for an alternative parameterization). Point estimates and confidence intervals/bands are based on 2,000 block bootstraps. **(A)** Population mean temperature response function for 4,730 maize hybrids. The solid line indicates the mean fixed effect coefficient function, and confidence bands at two confidence levels are shown. The vertical difference between any two points on the function indicates the percentage change in yield associated with substituting one hour of exposure at one temperature for another. **(B)** The left panel indicates the mean (dot) and 90% (thick line) and 95% (thin line) confidence intervals for selection on average yield (random hybrid intercepts, *l*_*i*_). The right panel illustrates selection on the breeding values for the temperature response functions. Figure elements have the same meaning as in panel **(A)**. **(C)** Mean correlation between average yield (random hybrid intercepts, *l*_*i*_) and temperature response function coefficients (*β*_*ih*_) for hybrids grouped by year of introduction. Labels indicate significance at the 90% (^) or 95% (*) confidence levels. **(D)** K-means clustering results for centered and scaled time series of weighted mean hybrid cohort temperature response function coefficients. Each curve represents the time series for response to a 1°C temperature bin smoothed by a cubic B-spline with seven internal knots.

The population mean coefficient function exhibits the same non-linear, negative effect of temperatures >30°C found by [9–11,27,28] but without specifying an optimal temperature range (Fig 2A and S11A Fig), and these negative effects are significant for temperatures ≥33°C (95% bootstrapped confidence level). The vertical distance between any two points on the curve indicates the percentage change in yield when substituting one hour of exposure to one temperature with one hour of exposure to another temperature. For example, substituting a one-hour exposure to 30°C with a one-hour exposure to 37°C predicts an approximately 0.21% reduction in yield.

To reduce potential biases in the estimated strength and direction of selection [47], breeding values must be derived from a model that appropriately specifies common environmental effects [37], which can otherwise be absorbed into the estimated breeding values [47,48], leading to false positive genetic trends. Alternatively, common environmental effects can absorb genetic trends, reducing the power to detect selection [47]. Separation of these trends is further complicated when individuals are observed in a subset of years, leading to absorption of genetic by environmental trends [48]. The main output of the yield trials in our dataset are 2- and 3-year average yields to help farmers select stable, high-yielding hybrids. Consequently, 1,727/4,730 (39.5%) of hybrids are tested in only 2–3 years. However, this implies that the majority of hybrids are tested in at least four years and does not account for the fact that genetically-identical hybrids can be grown in multiple locations unlike the studies of selection in natural populations reviewed by [37,48]. The ability to separate genetic and environmental trends in such situations is dependent on the distribution of hybrids across years and the degree of genetic relatedness between pairs of hybrids [49] when both are considered as random effects. Although we lack pedigree and marker data for these hybrids, we predict the variances of contrasts between different hybrids or years under assumptions that would generate the largest theoretical variances [49,50] and show that these are, in general, small, indicating a good degree of connectivity even in the absence of pedigree data (S12 Fig).

Directional selection on breeding values is then estimated by regressing cohort mean breeding values on time [51]. Additionally, tests of selection by regression are anticonservative due to the non-independence of breeding values in the mixed model [51]; thus, uncertainty in the estimated strength and direction of selection is assessed in this study using a block bootstrap as previously described.

Our model estimates breeding values for both average yield (random hybrid intercepts) and temperature response function coefficients (see Eq (1), “Methods”) to capture the genetic components of increasing average maize yields and heat tolerance, respectively. To estimate selection on these breeding values, hybrids are assigned to the year in which they first appear in the dataset. A regression coefficient whose confidence interval excludes zero indicates that the mean breeding value has changed [47,51]. The estimated selection coefficients are depicted in Fig 2B (S11B Fig) for average yield (left sub-panel) and temperature response functions (right sub-panel) at the 90% and 95% confidence levels.

First, we find evidence of directional selection on hybrid intercepts at a mean rate of 0.32% per year [95% CI (0.05, 0.71)], consistent with increases in *per se* yields during the 20th century [1]. Second, we find evidence at the 90% confidence level that selection has been acting to increase resistance to moderate heat stress caused by temperatures of 32–34°C over the previous 81 years. This agrees with numerous reports of improvements in drought-adaptive traits during the 20th century summarized by [2]; previous evidence for trends in heat tolerance using different datasets, models, and assumptions [27]; and recent, explicit selection for tolerance to these stresses in commercial breeding programs [52]. Third, this beneficial selection is counterbalanced by evidence for a *decrease* in resistance to severe heat stress caused by temperatures >38°C at a rate approximately one order of magnitude greater than the rate of improvement to moderate heat stress, consistent with the trends observed by [27] and the implications of the predictions made by [28]. The results using a constant B-spline basis are qualitatively similar, estimating trends in the same directions but with increased uncertainty (compare S11A, S11B and S13 Figs).

### Increases in sensitivity to severe heat stress despite favorable environmental trends

Maize development is temperature-dependent [20], and the effects of different environmental factors on phenotype have been shown to vary across developmental periods in multiple species, including maize [28,53–59]. Thus, changes to phenology that shift the developmental exposure of heat stress—particularly to the susceptible flowering and grain-filling periods—may lead us to overestimate the negative effects of high temperatures and the strength and direction of any trends in breeding values for response coefficients, especially if exposure to stressful temperatures has also increased.

While the trial records in our study lack flowering time data, we observe trends toward earlier planting dates in most of the 138/172 counties with at least four years of data across 1934–2014 (S14A Fig), which is associated with a small, but non-significant, negative effect on log-yield in both model specifications (S7 Fig). These trends, however, are associated with significant *increases* in exposure to optimal temperatures and *decreases* in exposure to stressful temperatures in most counties (S14B Fig). Despite rising temperatures at the global level [3], the US Corn Belt is at the center of a “warming bubble” [60,61], where average summer temperatures have decreased, leading to a (temporarily) more favorable growing environment for maize.

We can examine the potential impacts of phenological shifts on the developmental timing of heat stress by using state-wide crop progress data collected by the USDA for 1982–2014. We used a logistic model following [62] to estimate the timing of developmental stages for each year’s crop and the method of [59] to estimate stage-specific exposure distributions for 74/172 counties with at least four years of data from 1982–2014. There were few significant trends toward increased heat stress exposure during the grain-filling period (S15 Fig) in agreement with the previous analysis.

Overall, there is evidence that the growing environment has become more favorable to maize in our dataset. Because the tendency of the mixed model is to absorb genetic differences into environmental trends when individual genotypes are observed in a subset of years as is the case in our dataset [48], this strengthens the evidence for real genetic change underlying trends in average yields and moderate heat stress tolerance in addition to favorable environmental trends. Similarly, the observed genetic trend toward less severe heat stress tolerance is occurring in the presence of (a) favorable environmental trends and (b) increasing average yields, which may be an instance of cryptic evolution—genetic change masked by environmental change. (See Fig 3 of [47] for recommendations on the interpretation of such trends.)

**Fig 3:**
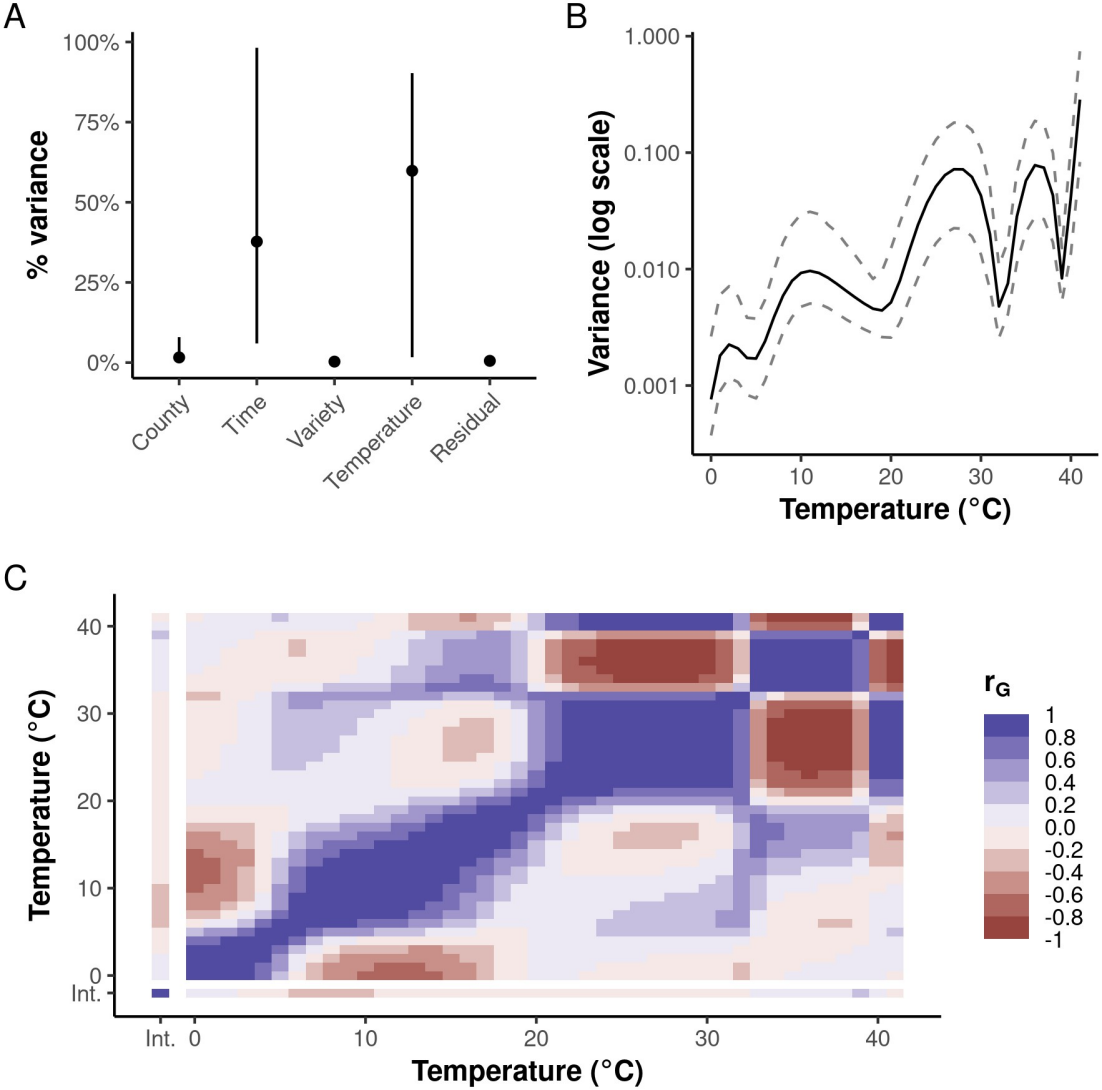
Variance decomposition of hybrid trial yields. Results shown are based on 2,000 block bootstraps for the model where exposure distributions and response functions are parameterized by cubic B-splines (see “Methods” and S17 Fig for an alternative parameterization). **(A)** Percentage variance attributed to random effects. “Variety” indicates the genetic variance in hybrid intercepts; “Temperature,” the combined genetic variance for responses to all temperatures. Points indicate means and lines the 95% confidence intervals. **(B)** Genetic variance of breeding values for temperature response functions (N.B. the logarithmic scale). The solid line indicates the mean; dashed lines, the 95% confidence bands. **(C)** Genetic correlation function for the correlation between breeding values for temperature responses at different temperatures and the hybrid intercepts (bottom-most and leftmost row and column, respectively). “Int.” indicates the hybrid intercept. The bootstrapped mean function is depicted.

### Temperature responses are weakly and variably correlated with hybrid intercepts

Because we observe evidence for selection in both hybrid intercepts and some regions of the temperature response function, a reasonable hypothesis is that change in the latter is the result of correlations to the former. We therefore calculated the correlation between the hybrid intercept and temperature coefficients within each hybrid cohort (13–123 hybrids/cohort; Fig 2C and S11C Fig). This revealed generally weak, non-significant, and fluctuating correlations across the time period of our dataset. This suggests that trends in temperature responses are unlikely to be an indirect response to selection on hybrid intercepts.

### Trends for responses to moderate and severe heat stress are complex but anticorrelated

While we observe small directional trends across 1934–2014 for responses to temperatures >30°C, [27] previously observed a fluctuating trend for the combined effect of temperatures >29°C with a maximum near 1960. We focused on the region of the temperature response functions for heat stress (>30°C) and calculated weighted mean temperature response coefficients for each year. To identify common temporal patterns of selection across coefficients, we applied k-means clustering to the historical trajectories of these temperature-specific, mean coefficients. This identified three clusters (Fig 2D), two of which included non-zero domains of the selection function (Fig 2B). This revealed that temperature responses to 30–35°C and 37+ °C have been changing in anticorrelated fashion throughout the 20th century, leading to multiple local maxima and minima that alternate between the two primary clusters. Such a pattern suggests the influence of other evolutionary forces on heat stress responses in hybrid maize, including, but not limited to, genetic drift, changing selection pressures, indirect selection from other fitness components, and genetic tradeoffs internal to temperature response mechanisms.

The trajectories in Fig 2D also suggested that the strength and/or direction of selection changed around 1975. We repeated the selection analyses, separating hybrids into those introduced before and after 1975 (S16 Fig). The estimated direction of selection did not change for the cubic B-spline model, although it did indicate a trend toward stronger selection post-1975. The constant B-spline model, however, now estimated statistically significant evidence for selection, especially for responses to temperatures >37°C, that switched direction after 1975. In particular, this model estimated a *decrease* in sensitivity to such temperature pre-1975 and an *increase* in sensitivity to such temperatures post-1975. This switch could provide a more fine-grained characterization of the trend observed by [27].

### Sources of variation for yield and temperature response functions

Considering the wide range of predicted outcomes for maize yields under climate change and ongoing plant breeding efforts to improve drought and heat tolerance, it is important to assess the potential for genetic adaptation to different temperatures by examining patterns of genetic covariation for temperature responses, which will constrain the directions in phenotypic space that are most genetically accessible. Variance for log-yield in this dataset is mostly attributable to the effects of changing agronomic practices over time and the interaction of genotype and environment captured by the functional regression on temperature exposure (Fig 3A). The amount of genetic variation for responses to different temperatures varies over three orders of magnitude in our historical dataset and is highest for temperatures >20°C (Fig 3B). This agrees with our earlier observations that directional selection on responses to extreme temperatures has been weak (Fig 2B, right panel) and likely not under significant indirect selection pressure from the much stronger selection on hybrid intercepts (Fig 2B [left panel] and **C**) during 1934–2014. Therefore, we would expect that if genetic variance for responses to stressful temperatures exists, it would likely be large relative to genetic variance for optimal temperatures, for example. However, we see that the amount of genetic variance for temperatures >20°C is relatively high, which suggests the presence of unused genetic variation for more beneficial responses to both optimal and stressful temperatures.

However, selection acting on any continuous region of the temperature response function is also subject to genetic constraints between different regions of the function. The genetic correlation function describes the relative strength of the genetic covariance between responses to different temperature exposures and is the infinite-dimensional analogue of the genetic correlation matrix estimated in multivariate selection studies [40,63]. We observe strong, positive, local correlations between responses to similar temperatures along the diagonal (Fig 3C). Similar to the anticorrelation for time-series temperature response coefficients (Fig 2D), we also observe two negatively correlated regions corresponding to 33–39°C and 40+ °C (Fig 3C). These domains overlap—but are not identical with—the domains of the temperature response function experiencing different directions of selection (Fig 2B, right panel) and clusters of similar mean trajectories over time (Fig 2D) described above. Collectively, these suggest the existence of a genetic tradeoff for responses to moderate and severe heat stress (provisionally 30–35°C and 37+ °C, respectively).

Although there are other, weaker possible tradeoffs suggested by the genetic correlation function (e.g., 0–4°C vs. 6–18°C, Fig 3C), the tradeoff suggested for extreme temperatures is quite strong. We do not believe that this is an artifact of the model. First, the alternative specification (constant B-spline basis) estimates correlations that are generally indistinguishable from zero except along the diagonal (S17C Fig). That pattern is most likely due to the lack of continuity conditions at the knots due to the low order of the basis functions. However, the strength of the negative correlations increases in regions corresponding to those also predicted to harbor a genetic tradeoff by the cubic B-spline basis. Second, analyses of multivariate genetic correlations typically identify a much smaller number of effective dimensions of genetic variation, indicating the presence of genetic constraints on the total genetic variance observed across all studied traits [42]. This pattern also seems to hold for function-valued (or infinite-dimensional) traits like the temperature response functions studied here [42]. Third, moderate-to-strong tradeoffs between regions of the domains of other function-valued traits have been observed in other species [40,41].

### Complex modes of genetic variation for temperature response functions

**Fig 4:**
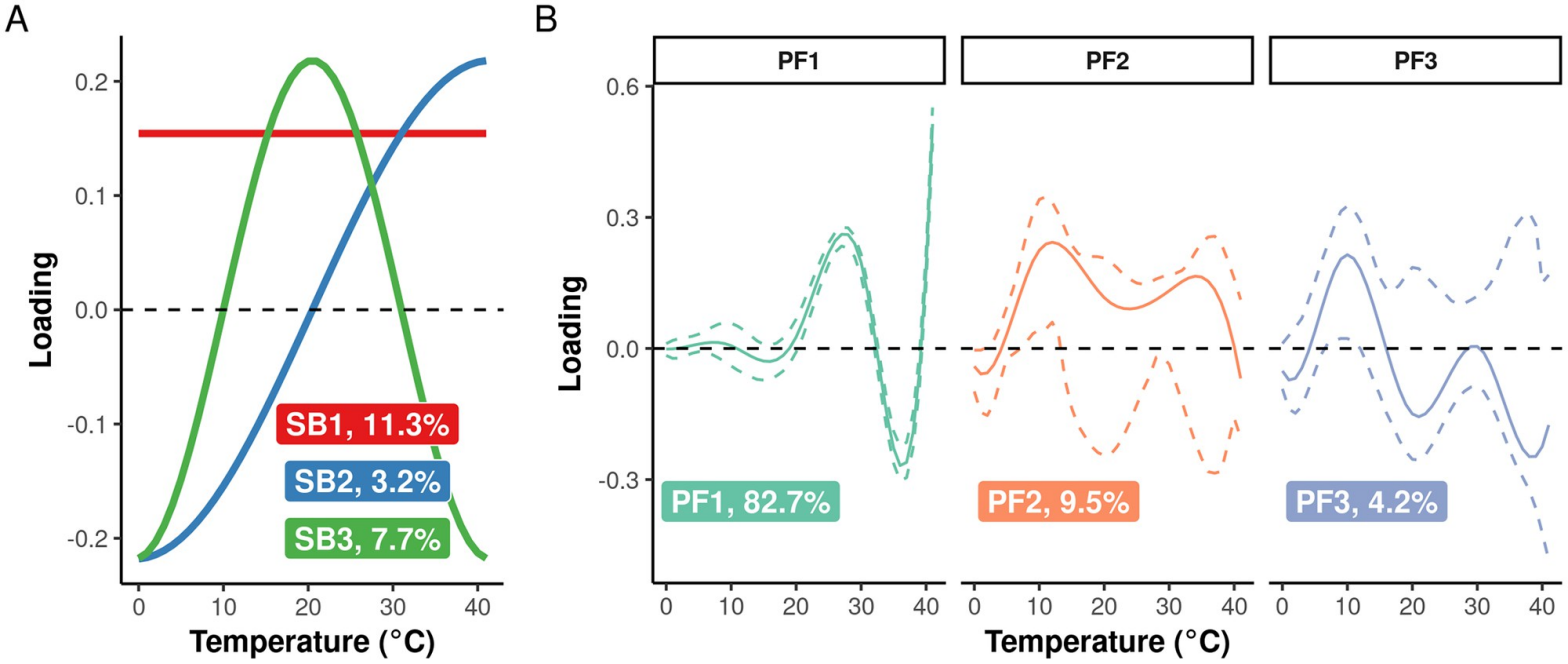
Modes of genetic variation for temperature response functions. Results shown are based on 2,000 block bootstraps for the model where exposure distributions and response functions are parameterized by cubic B-splines (see “Methods” and S18 Fig for an alternative parameterization). **(A)** Loadings for the first three simple basis (SB) functions of the genetic covariance function for temperature responses with the genetic variance explained by each function. The form of each SB function is determined by the choice of a simplicity metric in contrast to principal function analysis (PFA), which identifies functional responses of greatest variance. Each SB function describes genetic variation in a biological interesting direction: SB1 quantifies overall performance (i.e., log-yield); SB2 quantifies the strength of a tradeoff between low and high temperatures; and SB3 quantifies a generalist-specialist tradeoff. The percentage of genetic variance associated with each SB function is the bootstrapped mean. **(B)** Loadings for the first three principal functions (PF) of the genetic covariance function. These three PFs account for at least 95% of the total genetic variance in temperature responses. Solid lines indicate the bootstrapped mean; dashed lines, the 95% confidence bands. The percentage of genetic variance associated with each PF is the bootstrapped mean.

We used simple basis analysis (SBA) to obtain biologically interpretable modes of variation in the genetic covariance function [64,65] and the corresponding amounts of variance in these directions. Whereas principal functions analysis (PFA) identifies directions of maximal genetic variation, SBA optimizes a simplicity metric to calculate the genetic variation in predetermined directions in phenotypic space. Following the recommendations of [64], the first three SB functions for the genetic covariance function correspond to three classical biological modes of variation (Fig 4A) and capture 22.2% of the total genetic variance.

The first SB function is a horizontal line and measures variation for overall better performance (i.e., more positive responses to optimal temperatures and less negative responses to stressful temperatures) among hybrids. This direction accounts for 11.3% [(5.2, 18.0%), 95% bootstrapped confidence interval] of the total genetic variance. The second SB function measures a tradeoff between responses to temperatures less than or greater than 20°C—hybrids that are well-adapted to cold stress but not heat stress and vice versa. It captures 3.2% [(1.6, 5.6%), 95% CI] of the total genetic variance. Finally, the third SB function changes sign at 10 and 31°C, describing a generalist-specialist tradeoff, where hybrids that perform well at intermediate temperatures would be expected to perform poorly when exposed to extreme temperatures in either direction and vice versa. This direction accounts for 7.7% [(4.5, 11.5%), 95% CI] of the total genetic variance. SBA for the constant B-spline model accounts for a smaller proportion—12.8%—of the total genetic variance (S18A Fig).

Overall, these three SB functions accounted for less than 25% of the total genetic variance. We therefore performed PFA to compare the directions of maximal genetic variance for temperature response functions with those calculated by SBA. The first three principal functions (PFs) of the genetic covariance function accounted for 96.5% of the total genetic variance with 82.7% [(65.6, 91.9%), 95% CI] of the total genetic variance in the direction of the first PF alone (Fig 4B). Unsurprisingly, the first PF captures the strong, alternating patterns of correlation between responses to temperatures >20°C (Fig 3C). The second and third PFs are less amenable to interpretation, especially because a large degree of uncertainty makes it difficult to draw conclusion about the relative signs of the loadings. But in any case, they account for only 13.7% of the total genetic variation.

## Discussion

Maize yields have increased throughout the 20th century due to changes in both genetics and management practices [2]. Global climate change, however, threatens to impose severe yield penalties on maize over the course of the 21st century [9,11], leading to global food shortages [8]. Genetic adaptation to heat stress will be crucial to maintaining and increasing yields. Previous studies have often not addressed the question of genetic adaptation or found no evidence using proxy measures [9,27,31] [one exception is [66] but see the critique of [67]]. Large-scale panel studies in the US typically rely on aggregated county-level yields collected by USDA-NASS, where each data point represents a genetically heterogeneous mixture of hybrids that varies spatially and temporally. Thus, adaptive trends in genetics and management practices are confounded, leaving open the question of plant breeding’s realized and possible contributions to past and future temperature adaptation.

As a step towards answering this question, we analyzed changes in temperature adaptation over 81 years of hybrid maize breeding (1934–2014) using data from public university extension service yield trials in the US Corn Belt and daily temperature records. In contrast to earlier studies, this dataset enables estimation of temperature response functions for individual hybrids. We extended the model of [9] to estimate temperature response functions for 4,730 maize hybrids. This two-way panel data model [36] separated genetic changes due to direct or indirect selection on temperature response (Fig 2B) from location effects common to all hybrids grown in a particular county (S9 Fig) and time-dependent trends in management (S10 Fig) common to all hybrids grown in a particular state and year [30,68], allowing us to profile the course of genetic adaptation to temperature [33,38].

We were able to show that maize has been, at least indirectly and partially, genetically adapted to heat stress. In particular, for temperatures 32–34°C, this selection was in an adaptive direction at the 90% confidence level. This result is consistent with evidence for improvements in general abiotic stress tolerance and leaf rolling under drought in newer hybrids [2]. However, we also found evidence for a maladaptive shift in responses to extremely stressful temperatures (>38°C), which can cause yield penalties through kernel abortion, dry matter partitioning changes, and oxidative stress among other processes [69,70]. This result agrees with the observations of [27], the implications of the predictions of [28], and could provide a partial explanation for the increasing sensitivity to vapor pressure deficit observed by [14]. Nevertheless, we found that genetic variation for extreme temperature responses is high, suggesting the potential for future genetic adaptation to such temperatures (Fig 3B).

These shifts co-occurred with trends toward earlier planting dates and reduced exposure to heat stress (S14 and 15 Figs) as a consequence of the “warming bubble” over the US Midwest [60,61]. This phenomenon is attributed to agricultural intensification in the region during the twentieth century [71,72] and has produced a more favorable growing environment for maize [73]. Such a favorable environmental trend supports our conclusions of real and contrasting genetic trends for moderate and severe heat stress tolerance, following the recommendations of [47]. The maladaptive trend in severe heat stress tolerance may be a case of cryptic evolution, where environmental change masks genetic change, similar to the decrease in vapor pressure deficit tolerance observed in maize by [14]. This trend, in conjunction with the general increase in average maize yields, also suggests caution in the interpretation of the results of [17], who suggested that European maize yields may increase under climate change with appropriate phenological adaptation. However, as we have shown, phenological changes can mask genetic changes in other traits (here, heat tolerance) and lead to maladaptation under warmer environments despite “optimal” phenology.

We also documented fluctuating patterns of selection on heat tolerance across time (Fig 2D), which supports the hypothesis that heat tolerance has been under, at best, indirect selection mediated by genetic correlations with other traits and/or other evolutionary pressures. This also suggested changes in the strength/direction of selection over time, and an analysis of selection on temperatures >30°C pre- and post-1975, suggests that this may indeed be the case (S16 Fig). However, our two parameterizations gave evidence for differing trends in this analysis and warrant further investigation.

This inconsistent improvement in heat tolerance is counterintuitive when contrasted with the steady improvement in overall maize yields. However, this highlights the difference between yield *per se* and yield *stability*. While yield *per se* refers to the realized yield by a particular hybrid given some set of environmental conditions, yield stability refers to the variation around the *per se* value in a reference environment as environmental conditions vary. The heat tolerances (temperature response functions) that we estimated in this study are components of yield stability, not yield *per se*. Yield stability is a function of not only heat tolerance but resistance to drought, cold, pests, weeds, mineral availability, etc. Thus, as management practices change, shifts in limiting environmental factors also change selection pressures on their corresponding yield stability components, which can lead to counterintuitive trends such as increases in susceptibility to drought stress [12,14] or fluctuations in heat tolerance [27]. In short, [74] predicted such trends by noting that while modern maize hybrids are able to exploit favorable environmental conditions more fully, the accompanying greater year-to-year variability leads to larger decreases in yields (or, less yield stability) when environmental conditions are unfavorable. More recently, maize breeders have begun incorporating explicit selection on osmotic stress [52], which may modify these trends (similar to S16 Fig), although such hybrids were only widely introduced after 2014 (the final year of our dataset).

The temperature response functions estimated here are function-valued traits. Such traits generally have strong genetic constraints [42], and prediction of selection responses is complicated because the function—and not the response to any individual temperature—is the target of selection [40]. SBA of the genetic covariance function reveals that, in aggregate, three classical-biological modes of variation [65] account for approximately 22% of the genetic variance in temperature response functions (Fig 4A). By contrast, PFA demonstrates that the majority of the genetic variance resides in a different genetic subspace that imposes more complex shapes and a major constraint on temperature response evolution (Fig 4B; cf. Fig 3C). Because the non-linearities common to function-valued traits can cause non-intuitive responses to selection [40], direct selection for heat tolerance would need to account for the highly constrained shape of the genetic covariance function along with the potential to alter responses to optimal temperatures in unfavorable directions as a consequence of those constraints.

While we have leveraged a large dataset covering much spatial, temporal, and genetic variation, there are two avenues for further improvement. First, while we could estimate temperature response functions for individual hybrids, we lack pedigree or marker relationship data for these hybrids, which are generally unavailable. Incorporation of a relationship matrix into the estimation of the temperature response functions would increase the amount of information borrowing between them and help separate genetic from common environmental effects [37,48] by increasing the precision of the estimated differences between hybrids developed at different times [49]. This would be particularly useful for datasets like ours where each individual hybrid is observed in a subset of years (per the design of variety tests), which is a situation that increase the absorption of true genetic trends by environmental trends [48]. Second, we have assumed [following [9]] that the effects of temperature are identical over the growing season, which is manifestly false for maize. However, models making this assumption have proven robust in maize [9,27,43] and similar to estimates derived from process-based crop growth models [11]. Studies in wheat [33,34], sorghum [38], and maize [28,75] have demonstrated the utility of allowing time-varying temperature effects. Crucially, compared to our study, all of these studies reduce model complexity by some combination of constraining the functional form of the temperature response, admitting genetic variation for subsets of the temperature domain, or reducing the complexity of the exposure distribution. A model that combined the flexibility of ours with biologically appropriate, structural assumptions would be ideal but would also benefit from data on genetic relationships and observing each hybrid in a large number of environments. Ultimately, all of these studies draw on datasets collected as part of national statistics programs or designed for purposes other than estimation of heat tolerances *per se* and highlight the need for the development of datasets to study these questions explicitly.

In conclusion, combined with reports on commercial breeding programs [52], our findings promote a tempered optimism for the capacity of plant breeders to improve heat adaptation in the 21st century. Efforts over the past 80 years have, at least indirectly, led to some adaptive trends but also reveal underlying genetic tradeoffs. Whether or not these tradeoffs can be overcome and further adaptation achieved fast enough to match or exceed the rate of climate change remains an open question in quantitative genetics terms. However, because a typical modern maize hybrid takes <7 years to develop and commercialize [76] and multiple emerging technologies may combine to further reduce generation times [77], it may be possible for breeders to adapt maize hybrids sufficiently rapidly to respond to changes in climate. Genetic analyses of heat [78] and drought [79] tolerance have identified numerous mechanisms by which plants can adaptively respond to heat and osmotic stress. Targeting these mechanisms has been shown to improve drought tolerance in maize hybrids [52]. This and continued research combined with emerging technologies are expected to contribute to further, more targeted adaptations in the future [52,80,81].

## Materials and methods

### Yield data collection and curation

Historical maize performance data were collected from print and online publications produced by university extension programs in the US states of Illinois (IL), Iowa (IA), Kansas (KS), and Nebraska (NE). More than 75 years of publications were collected for each of IL, IA, KS, and NE. Reports from IL, IA, and KS were converted to editable text files using optical character recognition software (ABBY Fine Reader, v12.1.3) and manually entered into Microsoft Excel spreadsheets.

We used the Amazon Mechanical Turk (AMT) service to assist with conversion of the NE reports. Reports for 1948–2009 were obtained as low-quality scans of paper publications. A first campaign was run wherein AMT contractors were asked to enter data from the scans into Microsoft Excel spreadsheets manually. NE reports from 2010–2016 were of sufficient quality to be converted using ABBY Fine Reader and formatting errors were fixed by an automated Perl script. All NE spreadsheets were then submitted to a second AMT campaign to check the accuracy of data entry against the source PDFs. As a quality control measure, we introduced subtle changes to approximately 10% of the entries in a spreadsheet at random. A spreadsheet was considered checked if at least 70% of the introduced errors were identified and corrected. A bonus payment was disbursed if at least 90% of the introduced errors were identified.

Checked spreadsheets from all states and years were then manually curated to maintain consistent formatting, spelling, and capitalization of brand and hybrid names. Reports from all years and states were combined and irrigated trials were removed. The remaining records were filtered (1) on the basis of the connectedness of counties and years and (2) to include varieties that had been extensively tested. For the connectedness filter, we required each county to be represented in at least two years of trials and each year to be represented in at least two counties. Note that this latter filter did not remove any data. We also required each hybrid to have a minimum of 15 records which had to have been collected from at least two counties and two years.

Planting and harvesting dates were typically recorded for all trials. Trials missing either or both of these dates were excluded from further analysis. Due to regional variation in agronomic practices and weather, some trials were harvested as late as November or December. Therefore, calculations of temperature exposure and precipitation began on the reported planting date and ended on the earlier of the recorded harvest date or 30 September.

Trial locations were reported by the US county containing the trial site and the town nearest to the trial site. Some more recent trials also include GPS coordinates for trial locations. For locations without GPS coordinates, county and town names were manually checked for accuracy. These names were used to query the geocoding service at openstreetmap.org to obtain approximate GPS coordinates for all trial locations.

### Weather data

Weather data were collected and generated following [9,43] (S1 Fig). A dataset of daily predictions for minimum and maximum temperature and precipitation was constructed using the 4x4 km grid PRISM monthly dataset [82–84]. Only those PRISM grids that intersected with counties where a trial was located were retained. GPS coordinates for county boundaries were obtained from the US Census Bureau (https://www2.census.gov/geo/tiger/TIGER2017/COUNTY/tl_2017_us_county.zip). Monthly PRISM records were supplemented with denser, daily weather records from the US National Weather Service (NWS) Global Historical Climate Network (GHCN) stations. Stations were removed if they had moved more than 0.035° of latitude or longitude.

PRISM grid cells were linked with the seven nearest GHCN stations having near continuous daily weather records for each year. A near continuous record is defined as a station having no more than three missing daily values for a given weather variable for the period January to September. Stations were linked separately for each variable such that a station could be linked for daily maximum temperature but excluded on the basis of excessive missing values for daily precipitation, for example. Missing daily values for GHCN stations were imputed from the daily values at the seven closest GHCN stations with non-missing values on that day and half-month fixed effects (e.g., 1–15 May, 16–31 May, 1–15 June, etc.). Ordinary least squares was used to predict missing values for minimum and maximum temperature, and Tobit regression for precipitation [implemented in the R package ‘VGAM’ [85]]. A type I Tobit model was used to account for the left-censoring at zero in precipitation records because negative precipitation is unrealistic.

Regression models for each PRISM grid cell were constructed by regressing monthly PRISM values on monthly GHCN values derived from the seven closest stations in the imputed GHCN dataset for each variable as described above. These monthly relationships were then used to predict daily weather values at the resolution of the PRISM grid using the daily values from the seven closest GHCN stations. This produced a dataset of daily minimum and maximum temperature and precipitation covering the growing seasons of each trial at a 4x4 km resolution across all counties that contained trials.

We then approximated the distribution of temperatures across each day using a sinusoidal model [86] and calculated the time in hours that each grid cell was exposed to 1°C temperature bins on each day throughout the growing season. The exposure time to each temperature bin for a trial was calculated by summing the time exposed to each temperature over the growing season in each PRISM grid cell and taking the average of all grid cells that intersected with the trial’s county. Precipitation was calculated in a similar fashion.

Prior literature has used fixed growing seasons for estimating temperature exposure [e.g., [9,11]]. Because we have information on planting and harvesting dates for each trial in our dataset, we can more closely model the actual temperature distribution experienced by the hybrids [28]. Using a fixed growing season of April 24 (the date by which half of the trials were planted) to September 30 overestimates the length of the growing season by, on average, 374.4 h (15.6 d). Due to its greater biological relevance, we used a variable growing season and included a fixed regression coefficient on planting date in our model.

The final weather dataset was determined by retaining trials experiencing 250–800 mm of precipitation (inclusive) across the growing season. The temperature exposure distributions were left-censored at -1°C and right-censored at 41°C.

### Statistical model

To incorporate genetic variation into the estimation of the effect of temperature on maize yields, we extended the two-way panel data [36] model of [9,44] to a functional linear mixed effects model (FLMM; S2 Fig) [45]. In the following, we first present the common form of the FLMM before discussing two alternative specifications and the optimization of the representations for the functional coefficients and predictors. The common form of the FLMM considered here is

$$y_{ijst}=\mu +\beta_{d}d_{jst}+f_{\mu }\left(p_{jst}\right)+c_{j}+f_{s}\left(t\right)+l_{i}+\int_{0}^{41}\left(g_{\mu }\left(h\right)+g_{i}\left(h\right)\right)x_{jst}\left(h\right)\;dh+\varepsilon_{ijst}$$ (1)

where *y*_*ijst*_ is the natural logarithm-transformed yield of the *i*th hybrid grown in the *j*th county of the *s*th state in the *t*th year; *μ* is the population mean; *β*_*d*_ is the fixed effect of Julian planting date, *d*_*jst*_, for the *j*th county of the *s*th state in the *t*th year; *f*_*μ*_ (*p*_*jst*_) is the fixed effect coefficient function for the effect of precipitation in the *j*th county of the *s*th state in the *t*th year, where *f*_*μ*_(·) is modeled by a cubic B-spline basis with five basis functions; *c*_*j*_ is the random effect of the *j*th county with distribution $N\left(0,\sigma_{c}^{2}\right)$; *f*_*s*_(*t*) is the random regression on time for the *s*th state evaluated in the *t*th year, where *f*_*s*_(·) is modeled by a second-degree, orthogonal polynomial basis and its coefficients have distribution $N\left(0,D_{t}\sigma_{t}^{2}\right)$; *l*_*i*_ is the random hybrid effect with distribution $N\left(0,\sigma_{l}^{2}\right)$; *g*_*μ*_(*h*) is the fixed coefficient function for the effect of exposure to temperature *h* on yield; *g*_*i*_(*h*) is the random coefficient function for the response of the *i*th hybrid to temperature exposure (i.e., temperature response function) with distribution $N\left(0,D_{g}\sigma_{g}^{2}\right)$; *x*_*jst*_(*h*) is a functional predictor returning the time (in hours) of exposure to temperature *h* for hybrids grown in the *j*th county of the *s*th state in the *t*th year; and *ε*_*ijst*_ is an independent error term with distribution $N\left(0,\sigma_{\varepsilon }^{2}\right)$. The coefficient functions *g*_*μ*_(*h*) and *g*_*i*_(*h*) and the functional predictor *x*_*jst*_(*h*) were modeled by B-spline bases of equal dimension. Although temperature exposure distributions were left-censored at -1°C, the lower limit of integration in (1) is 0, reflecting the absorption of the lowest temperature bin by the intercept term.

#### Alternative specifications of temperature functions

The integral in (1) captures potential non-linearities in the relationship between temperature exposure and log-yield. The representation(s) chosen for the functional components within the integral define a trade-off between the accuracy of the approximation of the integral and model complexity, particularly in the case of the *g*_*i*_(*h*)’s, where the variance-covariance matrix of the coefficients has *M*(*M* − 1)/2 unique entries and *M* is the dimension of the chosen representation.

[9] introduced three alternative representations of the integral in (1) that have been used in subsequent literature to model this non-linear relationship. To facilitate comparisons between representations, we approximated the integral using B-spline bases of two different orders and dimensions:

- Step model: This model uses internal knots placed every 3°*C* to define a constant B-spline basis (order 1, *M* = 14). While this model is more flexible than the GDD model, its complexity is high. We use this dimension for consistency with prior work in maize using this model [9,11].
- Polynomial model: This model uses a variable number of internal knots (see next section) to define a cubic B-spline basis (order 4). This is similar to the representation using Chebyshev polynomials by [9]. Depending on the dimension of the basis, it can express similar or greater flexibility to that of the step model but at a lower complexity. To the best of our knowledge, polynomial models have not been the preferred model specifications in past research.

While the dimensions of the basis expansions for both models could be optimized, we consider that for the step model fixed by prior literature for comparative purposes.

#### Optimization of the cubic B-spline basis

To choose a basis expansion for the functional predictor *x*_*jst*_(*h*) that optimizes the tradeoff between accurate representation of exposure times at different trials and subsequent model complexity, we devised a hybrid strategy [46,87]. For each trial, we sequentially fit regression splines using cubic B-splines as the basis functions and a number of internal knots, $n_{k}\in \left\{0,\ldots ,\lfloor \frac{43-2}{2}\rfloor \right\}$, where 43 is the number of discrete temperatures for which exposure times were estimated and subtraction of two accounts for the unique boundary knots. Addition of an internal knot at step *s* was accepted if the reduction in the mean squared error (MSE) of the fit met the relative criterion [87]:

$$\left|{\text{MSE}}_{s}-{\text{MSE}}_{s-1}\right|<\theta \left|{\text{MSE}}_{s}\right|$$

The parameter *θ* governs the tradeoff between goodness of fit and complexity. The mode of the optimal internal knot numbers for each trial was chosen as the optimum for the basis expansion of all trials (or median of the modes in the case of ties) [46]. This procedure selected three internal knots (*M* = 5) as optimal across the 2,581 trials, which was robust to variation in *θ* (S6 Fig).

#### Model fitting and uncertainty estimation

To fit the FLMM, we used the ‘splines’ and ‘splines2’[88,89] packages to fit regression splines to the temperature exposure data and the ‘fda’ package [90] to compute the inner products of the functional coefficient and predictor bases. Basis expansions for *f*_*μ*_(·) were computed with the ‘splines’ package and for *f*_*s*_(·) using ‘poly()’. We fit the full model using the package ‘lme4’ [91].

To account for the effects of spatial covariation among trials within each year, we implemented a Bayesian block bootstrap using year as the blocking variable. We resampled the dataset 2,000 times and refit Eq (1) using both parameterizations described above. Point estimates in the text are the bootstrapped mean estimate, and confidence intervals/bands are the 95% bootstrapped confidence intervals/bands (unless otherwise stated) calculated using the ‘bca()’ function of the ‘coxed’ package [92].

### Data connectivity

When environmental effects are treated as random (Eq (1)), all possible contrasts between levels of a single factor are estimable, but the precision of the contrast depends on their distribution over levels of other factors [49]. We used the second suggested method of [49] to assess contrast variances between different common environmental effects in our model. This requires the estimation of a covariance matrix between the levels of each factor. We approximate these matrices as

- County: A spatial distance matrix using the Haversine distance between county centroids.
- Year: The expected covariance between years when modeled as a quadratic polynomial of time (as in Eq (1)).
- Trial (county-year): Kronecker product of the covariance matrices for county and year.
- Hybrid: A diagonal matrix with two along the diagonal. This simulates a variance-maximizing scenario, where the parents of each hybrid are assumed to be completely inbred [50], and hybrids are assumed to be unrelated.

### Quantifying selection

Selection on temperature response functions was assessed by regressing the coefficients of the temperature response functions on calendar year for each resample to generate the bootstrap distribution for the selection function on temperature responses at 1°C intervals [51]. The temperature response function for each hybrid was assigned to the earliest year in which that hybrid appeared in the dataset. We used the number of observations for each hybrid as weights in the regression analysis to account for the unbalanced nature of the dataset. Confidence intervals that exclude zero are considered evidence of selection.

To examine correlated changes over time in the responses to different temperatures, we calculated weighted mean coefficients for each hybrid cohort as defined above and performed k-means clustering on trajectories for responses to temperatures ≥30°C. The optimal number of clusters was chosen by plotting the between-cluster sum of squares for 2–6 clusters and applying the elbow method.

### Trends in phenology and exposure time

To examine changes in phenology and temperature exposure over time, we took the subset of 138 counties with at least four years of data and regressed planting date (in Julian days), harvest date (in Julian days), growing season length (the difference between harvest and planting dates), and time exposed to each 1°C temperature bin (in days) on year.

Because the effects of temperature vary with development, we also approximated stage-specific trends in temperature exposure using the methods of [59,62]. We obtained crop progress data for Illinois, Iowa, Kansas, and Nebraska in 1982–2014 from USDA-NASS (www.nass.usda.gov), removing years with fewer than five progress observations. Daily progress percentages were estimated by fitting a logistic model with the R function ‘nls()’[62]; if recorded data did not include 0% or 100%, we approximated the missing days using linear regression on the first (or last) recorded weekly interval. We could then estimate the fraction of the maize crop in a state-year combination at a particular stage by subtracting from its progress estimate the progress estimate of the following stage [59]. We then calculated weighted temperature exposure distributions for the 74 counties with at least four years of data from 1982–2014, where the weights were the fraction of the crop estimated to be in each stage on a given day. Quantification of trends was performed as described for the un-staged trial data.

### Decomposing the genetic covariance function

The genetic covariance function describes genetic covariation between breeding values at different values of a functional covariate and is the infinite-dimensional analogue of the multivariate genetic covariance matrix [40,63]. For our study, the genetic covariance function $G\left(h,h^{\prime }\right)$ describes the covariance between breeding values for the response to temperature *h* °*C* and breeding values for the response to temperature *h*′ °*C*. When *h* = *h*′, G returns the variance of the breeding values for the response to the indicated temperature. Following [40,45,93], we calculated the genetic covariance function as

$$G\left(h,h^{\prime }\right)=\phi \left(h\right)D_{g}\phi {\left(h^{\prime }\right)}^{\prime }$$

where ***ϕ***(·) are the B-spline basis functions for the appropriate specification evaluated at temperature *h* °*C* and *h*′ °*C*, and ***D***_*g*_ is the estimated variance-covariance matrix of the temperature response functions as described in “Statistical model.”

We performed simple basis analysis (SBA) [64] and principal functions analysis (PFA) using the ‘prinsimp’ package [94] following the recommendations of [65]. We examined the first three simple basis (SB) functions of the bootstrapped mean genetic covariance function using a simplicity metric based on first divided differences [equation 4.1 of [64]]. We also examined the minimum number of principal functions (PFs) required to explain ≥95% of the total genetic variance. We then performed SBA/PFA on each bootstrapped sample of the genetic covariance function to generate bootstrapped distributions for each function and its variance explained.

## Supporting information

S1 Fig Workflow for construction of the historical weather dataset. Time exposed to 1°C temperature bins and season-total precipitation were estimated for historical trials by combining daily weather records from the Global Historical Climate Network (GHCN) and PRISM project. Full details are described in subsection “Weather data” of the “Methods.”
(PDF)

S2 Fig Modeling and analysis workflow. The relationships between variables in the data, the functional linear mixed effects model, and subsequent analyses are shown along with references to main text and supporting figures for results. For full details on the notation of the model, see “Statistical model” in “Methods.” Details of the different analyses can be found in the appropriate subsections of the “Methods.”
(PDF)

S3 Fig Temporal trends in yield and yield variability by state for 1934–2014. Each point represents the indicated summary statistic of hybrid yields (bu/a) grown in all trials in that state and year. Best fit regression lines of the indicated statistic on time, 95% confidence bands, and regression equations are shown in each panel. Mean yields and yield standard deviations are increasing across time in all states. However, mean yields are increasing at least as quickly as yield standard deviations, leading to a decrease or no change in the coefficient of variation of yield across time.
(PNG)

S4 Fig Average exposure to heat stress by county for 1934–2014. Average percentage of the total growing season (recorded planting date to the earlier of the recorded harvest date or 30 September) hybrids were exposed to temperatures >30°C in each county that contained at least two yield trials for 1934–2014. The map was generated using the ‘maps’ R package (https://cran.r-project.org/package=maps).
(PNG)

S5 Fig Distribution of exposure to different temperatures across treatment factors. Each point indicates the number of levels of the factor indicated by the *y*-axis that were exposed to the temperature indicated on the *x*-axis. The maximum number of factor levels is given in the *y*-axis label.
(PNG)

S6 Fig Optimization of the cubic B-spline basis for exposure distributions. The optimal number of knots (boundary plus internal) for a cubic B-spline basis was determined for each trial (*n* = 2,581) by iteratively adding knots placed at quantiles until the mean squared error was not reduced by a predetermined proportion (*θ*). “NA” indicates that the threshold was not reached by the maximum number of 20 knots. “*” indicates the mode of the optimal numbers of knots.
(PNG)

S7 Fig Effect of planting date. Mean effect of Julian planting date on percentage yield for models with differing representations of temperature effects. Neither estimate is significantly different at the 95% (thick line) or 90% (thin line) confidence level. Estimates are based on 2,000 block bootstraps.
(PNG)

S8 Fig Effect of precipitation on yield. Effects of precipitation on percentage yield for models with differing representations of temperature effects. Solid lines indicate the mean and shaded areas 95% confidence bands for 2,000 block bootstraps.
(PNG)

S9 Fig Spatial distribution and magnitude of location (county) effects. **(A)** Mean percentage effect of each county from the cubic B-spline model. The map was generated using the ‘maps’ R package (https://cran.r-project.org/package=maps). **(B)** Comparison of mean location effects in models with differing representations of temperature effects. The dashed red line indicates equality. In both panels, the means of 2,000 block bootstraps are shown.
(PNG)

S10 Fig Year effects on percentage yield. Each panel depicts the effect of year on percentage yield for the indicated state relative to 1981, the mean trial year in the dataset. Effects were estimated as the random regression of natural log-transformed yield (bu/a) on an orthogonal, quadratic polynomial of trial year. Solid lines indicate the mean and shaded regions the 95% confidence bands of 2,000 block bootstraps.
(PNG)

S11 Fig Quantification of directional selection on temperature response functions. Results shown in this figure are for exposure distributions and response functions parameterized as constant B-splines (see “Methods” and Fig 2 for an alternative parameterization). Point estimates and confidence intervals/bands are based on 2,000 block bootstraps. **(A)** Population mean temperature response function for 4,730 maize hybrids. The solid line indicates the mean fixed effect coefficient function, and confidence bands at two confidence levels are shown. The vertical difference between any two points on the function indicates the percentage change in yield associated with substituting one-hour of exposure within one 3°C temperature bin for another. **(B)** The left panel indicates the mean (dot) and 90% (thick line) and 95% (thin line) confidence intervals for selection on the random hybrid intercepts. The right panel illustrates the selection function on breeding values for the temperature response functions. Figure elements have the same meaning as in panel **(A)**. **(C)** Mean correlation between random hybrid intercepts (*l*_*i*_) and temperature response function coefficients (*β*_*ih*_) for hybrids grouped by year of introduction. Labels indicate significance at the 90% (^) or 95% (*) confidence levels. **(D)** Centered and scaled time series of weighted mean hybrid cohort temperature response function coefficients. Each curve represents the time series for response to a 3°C temperature bin smoothed by a cubic B-spline with seven internal knots.
(PNG)

S12 Fig Predicted contrast variances for the difference between two levels of one factor when distributed over levels of a second factor. Each panel shows the distribution of predicted contrast variances for the difference between two levels of a factor when distributed over the levels of the other indicated factor. For example, the left side of panel **A** shows the contrast variances of counties when distributed over hybrids. The bin width is 0.05 in panels **A**-**C** and 0.1 in panel **D**. There are 172 levels of county; 81 levels of year; 4,730 levels of hybrid; and 2,581 levels of trial. See “Methods” for more details.
(PNG)

S13 Fig Evidence for directional selection on temperature response functions at various confidence levels. For the estimation of directional selection, see “Methods”. The direction is the sign of the mean slope of 2,000 block bootstraps. Significance is defined as the exclusion of zero by the (100 × *α*)% confidence interval of 2,000 block bootstraps as indicated on the *y*-axis.
(PNG)

S14 Fig Phenological and temperature exposure trends by state for 1934–2014. (**A**) Trends in Julian planting and harvest dates and growing season length by county. Only counties with at least four years of data are shown. Color indicates the sign of the slope of a linear regression of the indicated variable on year. Solid tiles indicate significance at an uncorrected *p* < 0.05 level. (**B**) Trends in temperature exposure time (in days) by county. Figure elements are the same as in panel **A**. Vertical dashed lines indicate the optimal temperature bounds for growing degree day calculations (10 and 30°C) and the moderate/severe heat stress breakpoint (36°C) identified in the main text.
(PNG)

S15 Fig Trends in stage-wise temperature exposure by state for 1982–2014. Only counties with at least four years of data are shown. Color indicates the sign of the slope of a linear regression of the indicated variable on year. Solid tiles indicate significance at an uncorrected *p* < 0.05 level. Vertical dashed lines indicate the optimal temperature bounds for growing degree day calculations (10 and 30°C) and the moderate/severe heat stress breakpoint (36°C) identified in the main text. Stages are defined as: “Vegetative” = planting to silking; “Early grain fill” = silking to dough; “Late grain fill” = dough to maturity; “Drydown” = maturity to harvest.
(PNG)

S16 Fig Directional selection on temperature response functions before and after 1975. The strength (cubic model) and direction (constant model) of selection differ between hybrids introduced before and after 1975. Results for the cubic B-spline parameterization are consistent in direction with those given in Fig 2B (right panel). Results for the constant B-spline parameterization show changes in direction for the different time periods and increased statistical significance compared with those given in S8B Fig (right panel). Solid lines indicate the mean strength and direction of selection, and dashed lines indicate the 95% confidence bands. Point estimates and confidence bands are based on 2,000 block bootstraps.
(PNG)

S17 Fig Variance decomposition of hybrid trial yields. Results shown are based on 2,000 block bootstraps for the model where exposure distributions and response functions are parameterized by constant B-splines (see “Methods” and Fig 3 for an alternative parameterization). **(A)** Percentage variance attributed to random effects. “Variety” indicates the genetic variance in hybrid intercepts; “Temperature,” the combined genetic variance for responses to all temperatures. Points indicate means and lines the 95% confidence intervals. **(B)** Genetic variance of breeding values for temperature response functions (N.B. the logarithmic scale). The solid line indicates the mean; dashed lines, the 95% confidence bands. **(C)** Genetic correlation function for the correlation between breeding values for temperature responses at different temperatures and the hybrid intercepts (bottom-most and leftmost row and column, respectively). The bootstrapped mean function is depicted.
(PNG)

S18 Fig Modes of genetic variation temperature response functions. Results shown are based on 2,000 block bootstraps for the model where exposure distributions and response functions are parameterized by constant B-splines (see “Methods” and Fig 4 for an alternative parameterization). **(A)** Loadings for the first three simple basis (SB) functions of the genetic covariance function for temperature responses with the genetic variance explained by each function. The form of each SB function is determined by the choice of a simplicity metric in contrast to principal function analysis (PFA), which identifies functional responses of greatest variance. Each SB function describes genetic variation in a biological interesting direction: SB1 quantifies overall performance (i.e., log-yield); SB2 quantifies the strength of a tradeoff between low and high temperatures; and SB3 quantifies a generalist-specialist tradeoff. The percentage of genetic variance associated with each SB function is the bootstrapped mean. **(B)** Loadings for the first three principal functions (PF) of the genetic covariance function. The first three PFs are shown, but the first nine PFs are required to account for at least 95% of the total genetic variance in temperature responses. Solid lines indicate the bootstrapped mean; dashed lines, the 95% confidence bands. The percentage of genetic variance associated with each PF is the bootstrapped mean.
(PNG)

S1 Table Geographical distribution of trial locations. Data underlying Fig 1A. Columns include American National Standards Institute (ANSI) county code, longitude (decimal), latitude (decimal), and total number of trials conducted in that county.
(CSV)

S2 Table Coefficients for the fixed effect of temperature on log-yield. Data underlying Fig 2A. Columns include temperature (°C), bootstrap, and estimate (% yield/h exposure).
(CSV)

S3 Table Slopes for the regression of random hybrid intercepts on time. Data underlying Fig 2B, left panel. Columns include temperature (“Intercept” to distinguish it from the data in S4 Table), bootstrap, and regression coefficient (% yield/yr).
(CSV)

S4 Table Slopes for the regression of random hybrid temperature coefficients on time. Data underlying Fig 2B, right panel. Columns include temperature (°C), bootstrap, and regression coefficient (% yield/h exposure/yr).
(CSV)

S5 Table Correlations between hybrid intercepts and temperature coefficients stratified by year. Data underlying Fig 2C. Hybrids were assigned to the first year in which they appeared in the dataset. Columns include bootstrap, temperature (°C), year, and Pearson’s correlation coefficient.
(GZ) Click here for additional data file.

S6 Table k-means clustering of cohort mean temperature coefficients. Data underlying Fig 2D. Cohort means are the weighted average temperature coefficient for all hybrids that first appeared in the dataset in the indicated year. Only temperature bins 30 to >41°C inclusive are included. Columns include year, temperature (°C), cohort mean coefficient, and cluster.
(CSV)

S7 Table Variance components. Data underlying Fig 3A. Columns include bootstrap, model component, and the estimated variance.
(CSV)

S8 Table Temperature variance function coefficients. Data underlying Fig 3B. Columns include temperature (°C), bootstrap, and estimated variance.
(CSV)

S9 Table Genetic correlation function for temperature. Data underlying Fig 3C. Columns include bootstrap, *x*-axis temperature (°C or “Intercept”), *y*-axis temperature (°C or “Intercept”), and Pearson’s correlation coefficient.
(GZ) Click here for additional data file.

S10 Table Variance explained by simple basis functions. Data underlying Fig 4A. Columns include bootstrap, simple basis function, and proportion of variance explained.
(CSV)

S11 Table Principal function loadings. Data underlying Fig 4B. Columns include bootstrap, principal function, temperature (°C), and principal function loading.
(CSV)

S12 Table Variance explained by principal functions. Data underlying Fig 4B. Columns include bootstrap, principal function, and proportion of variance explained.
(CSV)
