# Supplementary figures and images for: A genetic tradeoff for tolerance to moderate and severe heat stress in US hybrid maize
Source: PLoS Genet. 2023 Jul 6;19(7):e1010799. doi: 10.1371/journal.pgen.1010799 (PMC10325116; doi:10.1371/journal.pgen.1010799)

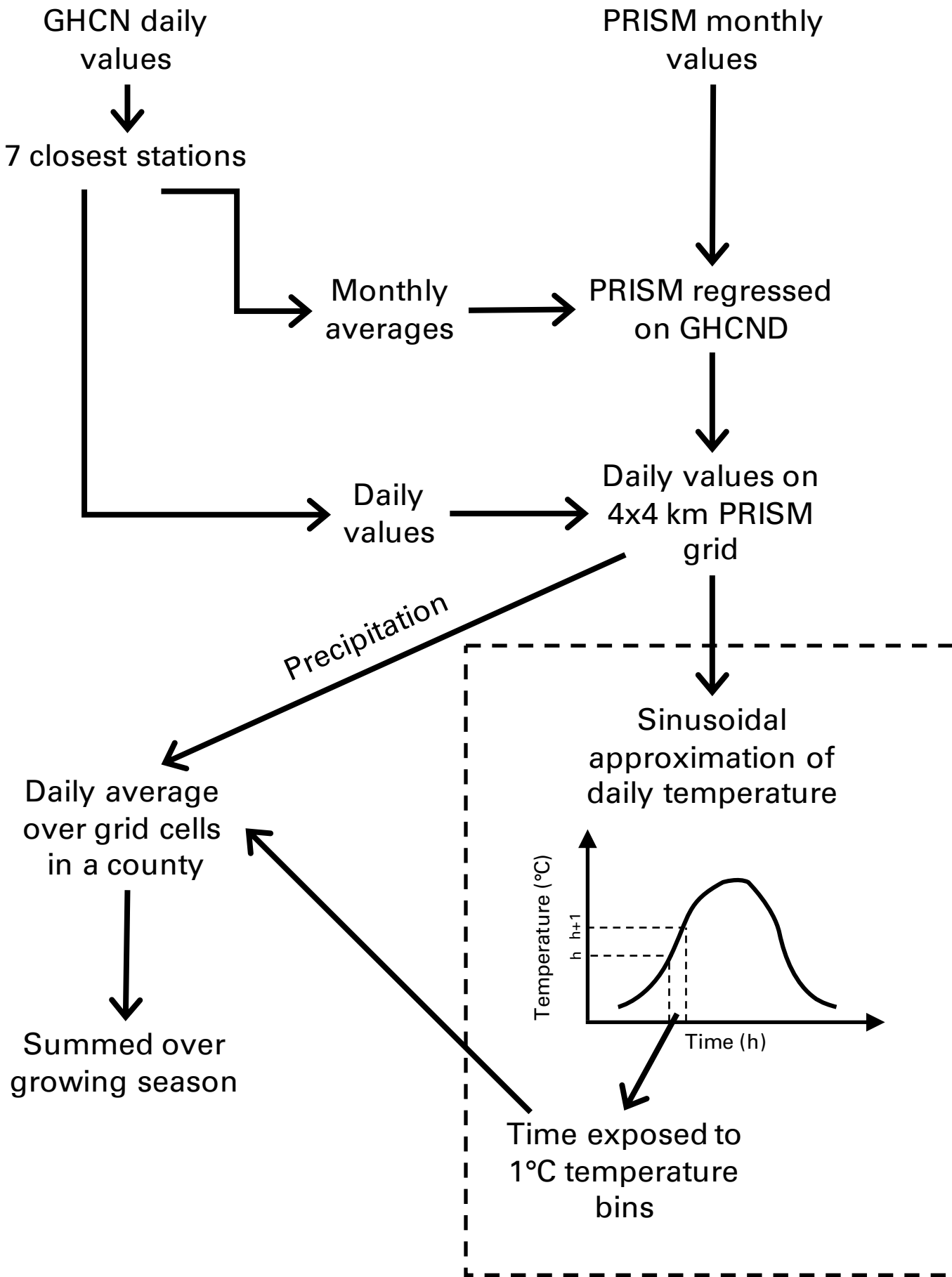

Supplement: S1 Fig — Time exposed to 1°C temperature bins and season-total precipitation were estimated for historical trials by combining daily weather records from the Global Historical Climate Network (GHCN) and PRISM project. Full details are described in subsection “Weather data” of the “Methods.” (PDF) [file pgen.1010799.s001.pdf]

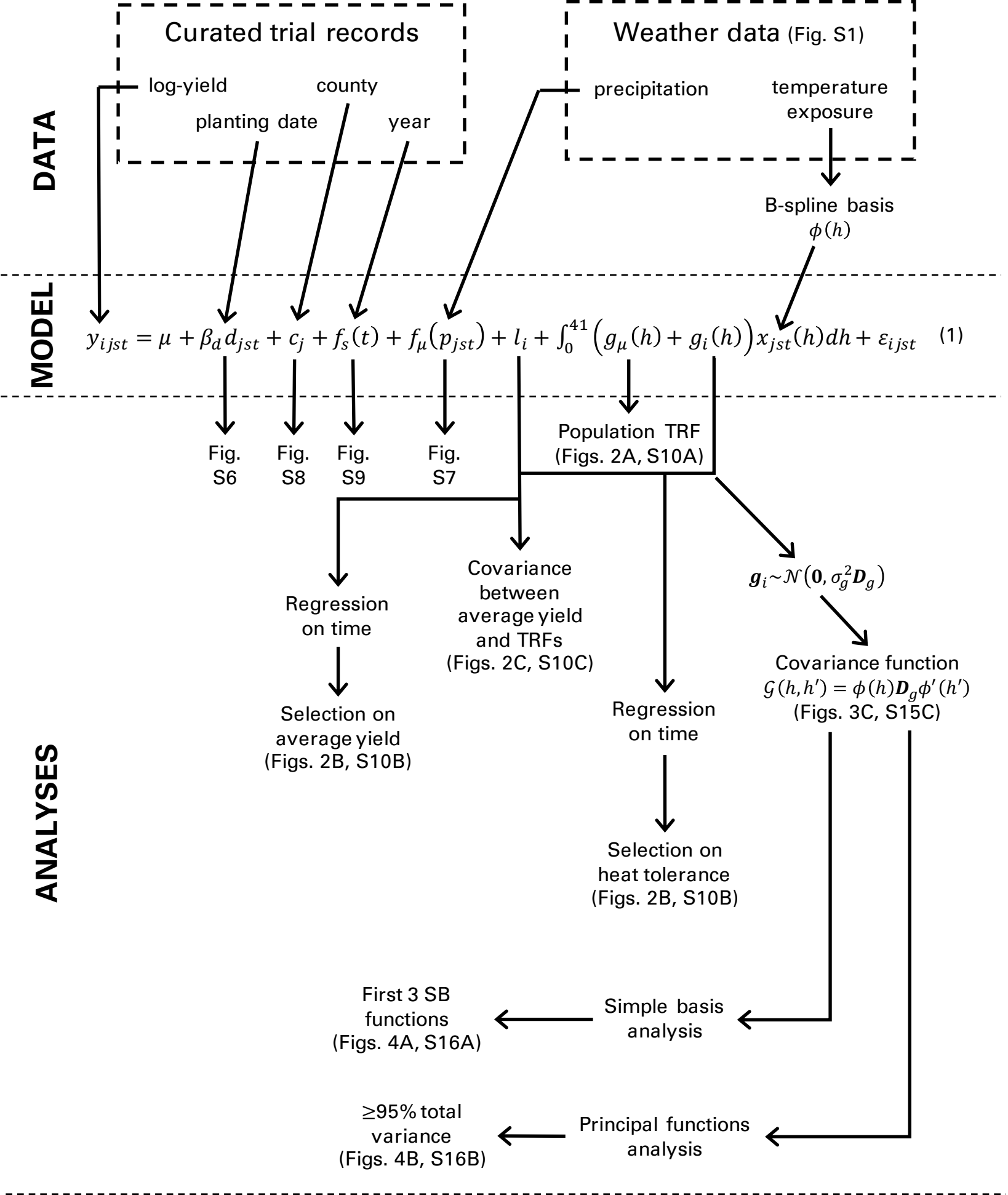

Supplement: S2 Fig — The relationships between variables in the data, the functional linear mixed effects model, and subsequent analyses are shown along with references to main text and supporting figures for results. For full details on the notation of the model, see “Statistical model” in “Methods.” Details of the different analyses can be found in the appropriate subsections of the “Methods.” (PDF) [file pgen.1010799.s002.pdf]

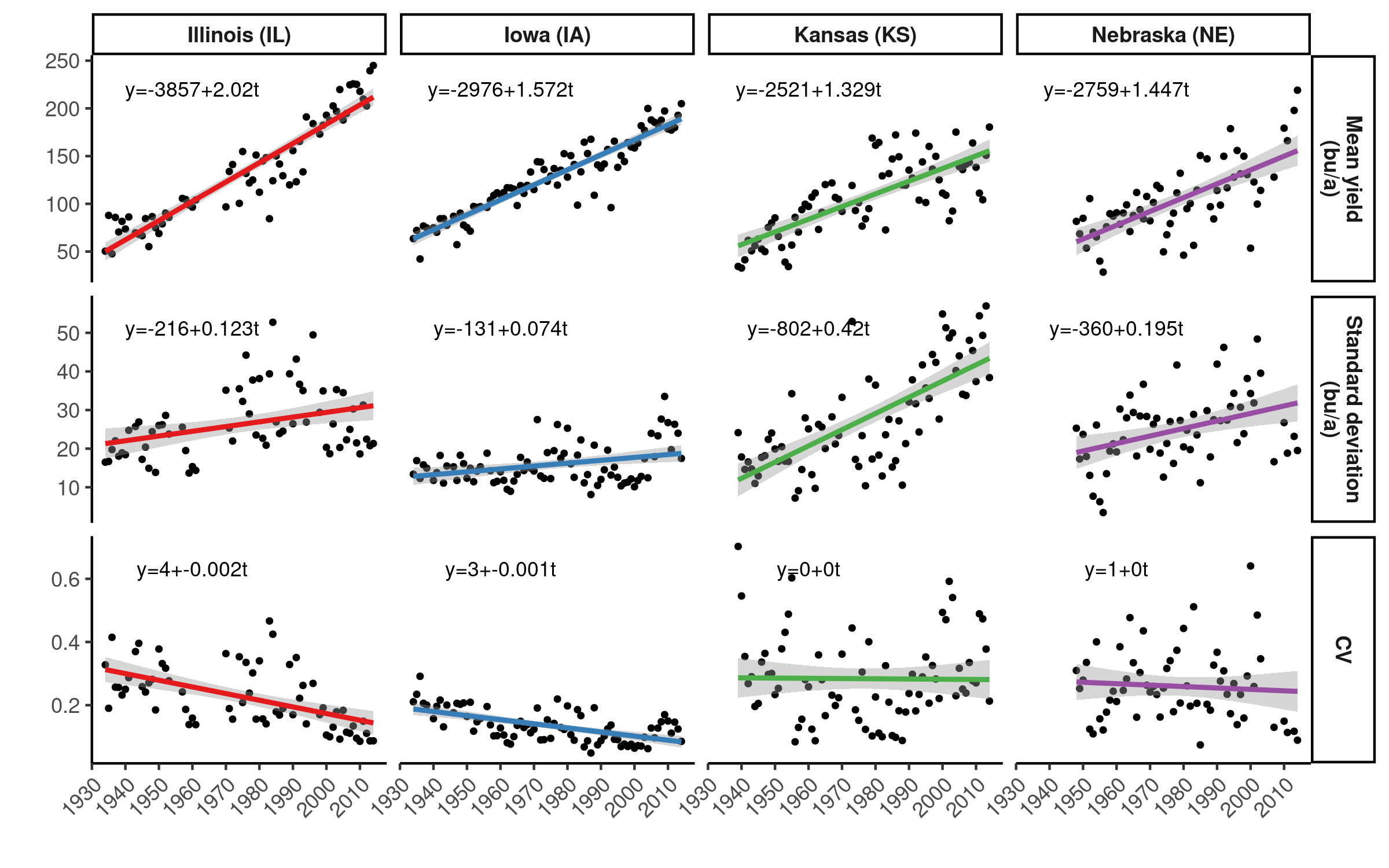

Supplement: S3 Fig — Each point represents the indicated summary statistic of hybrid yields (bu/a) grown in all trials in that state and year. Best fit regression lines of the indicated statistic on time, 95% confidence bands, and regression equations are shown in each panel. Mean yields and yield standard deviations are increasing across time in all states. However, mean yields are increasing at least as quickly as yield standard deviations, leading to a decrease or no change in the coefficient of variation of yield across time. (PNG) [file pgen.1010799.s003.png]

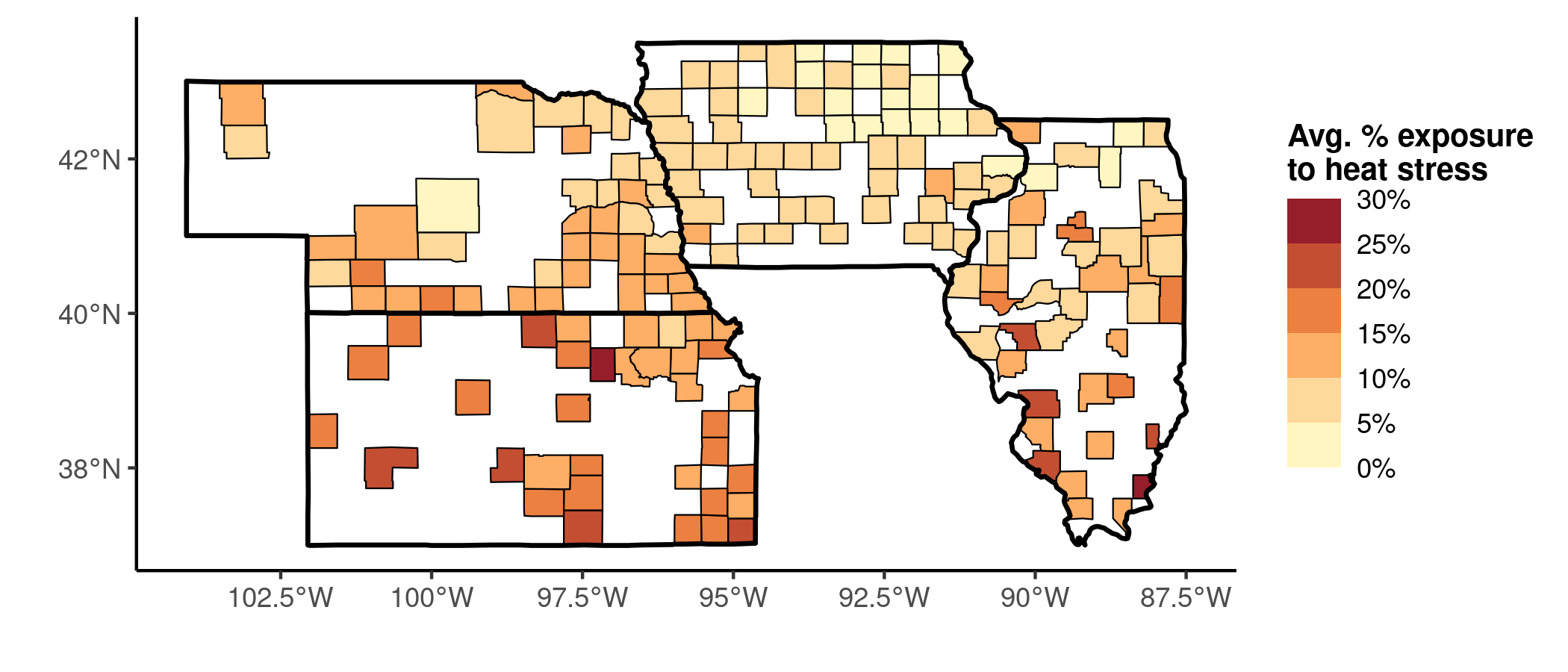

Supplement: S4 Fig — Average percentage of the total growing season (recorded planting date to the earlier of the recorded harvest date or 30 September) hybrids were exposed to temperatures >30°C in each county that contained at least two yield trials for 1934–2014. The map was generated using the ‘maps’ R package (https://cran.r-project.org/package=maps). (PNG) [file pgen.1010799.s004.png]

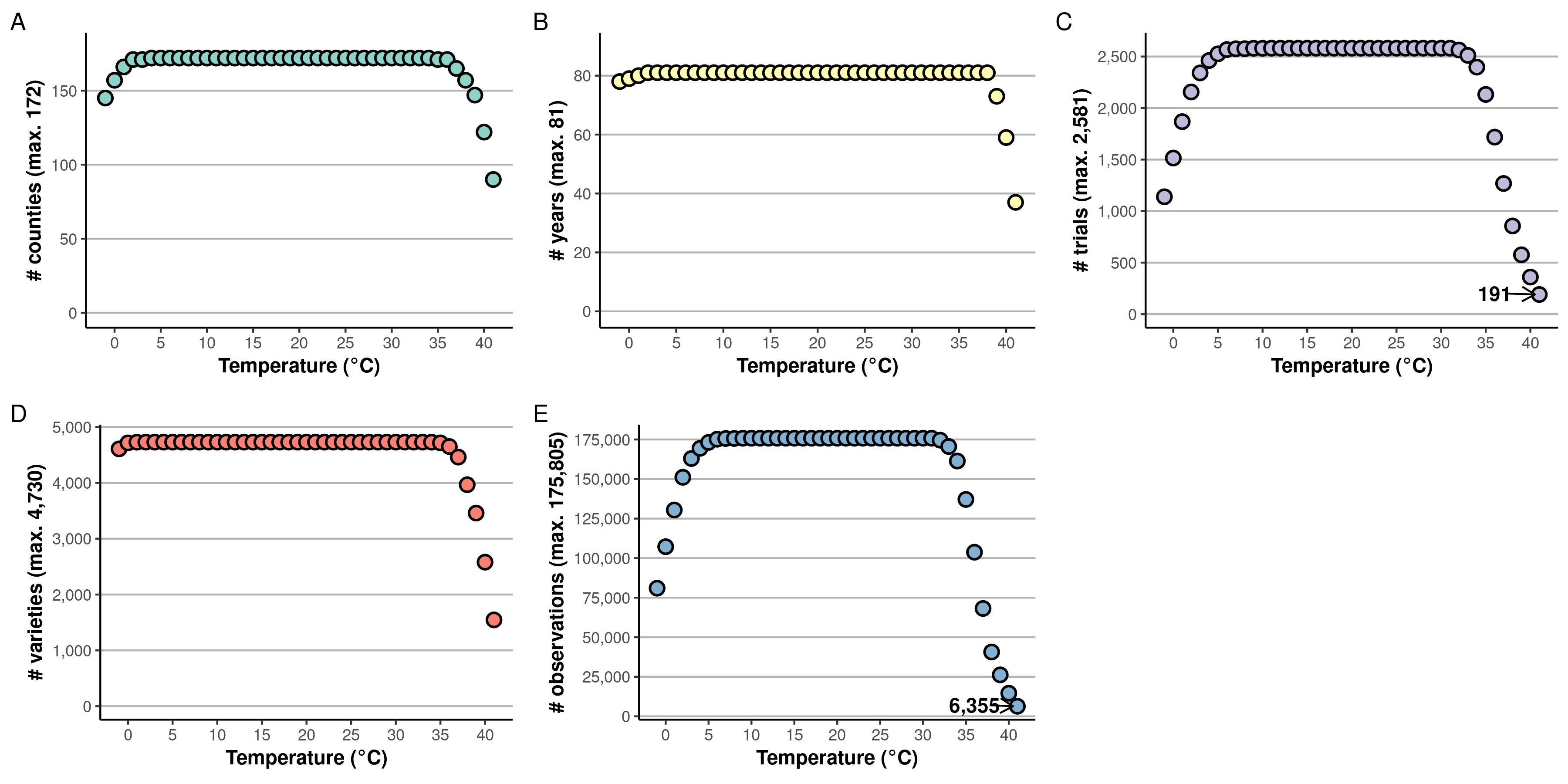

Supplement: S5 Fig — Each point indicates the number of levels of the factor indicated by the y-axis that were exposed to the temperature indicated on the x-axis. The maximum number of factor levels is given in the y-axis label. (PNG) [file pgen.1010799.s005.png]

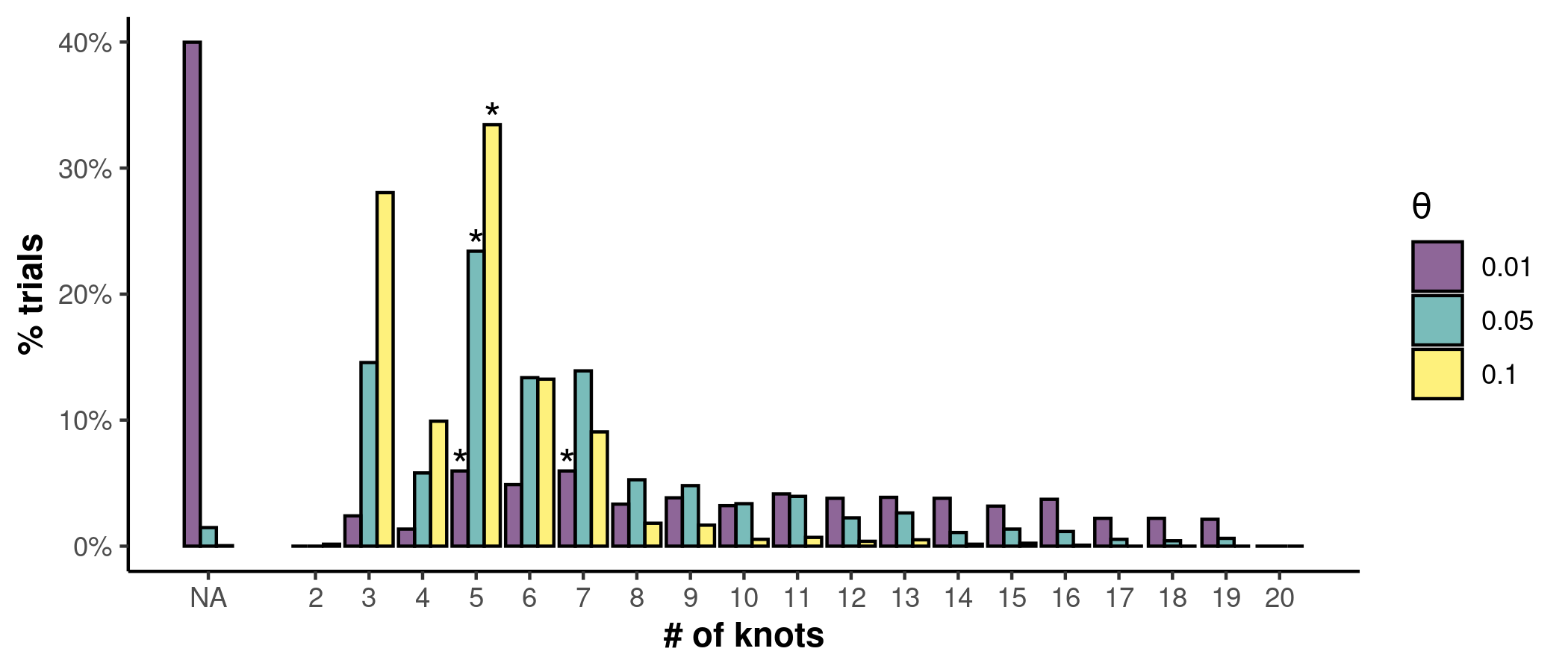

Supplement: S6 Fig — The optimal number of knots (boundary plus internal) for a cubic B-spline basis was determined for each trial (n = 2,581) by iteratively adding knots placed at quantiles until the mean squared error was not reduced by a predetermined proportion (θ). “NA” indicates that the threshold was not reached by the maximum number of 20 knots. “*” indicates the mode of the optimal numbers of knots. (PNG) [file pgen.1010799.s006.png]

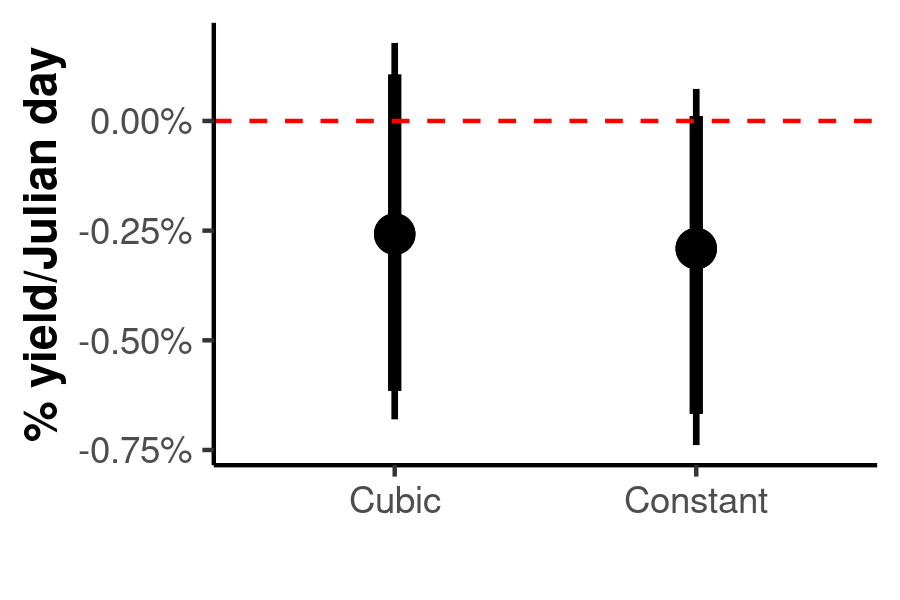

Supplement: S7 Fig — Mean effect of Julian planting date on percentage yield for models with differing representations of temperature effects. Neither estimate is significantly different at the 95% (thick line) or 90% (thin line) confidence level. Estimates are based on 2,000 block bootstraps. (PNG) [file pgen.1010799.s007.png]

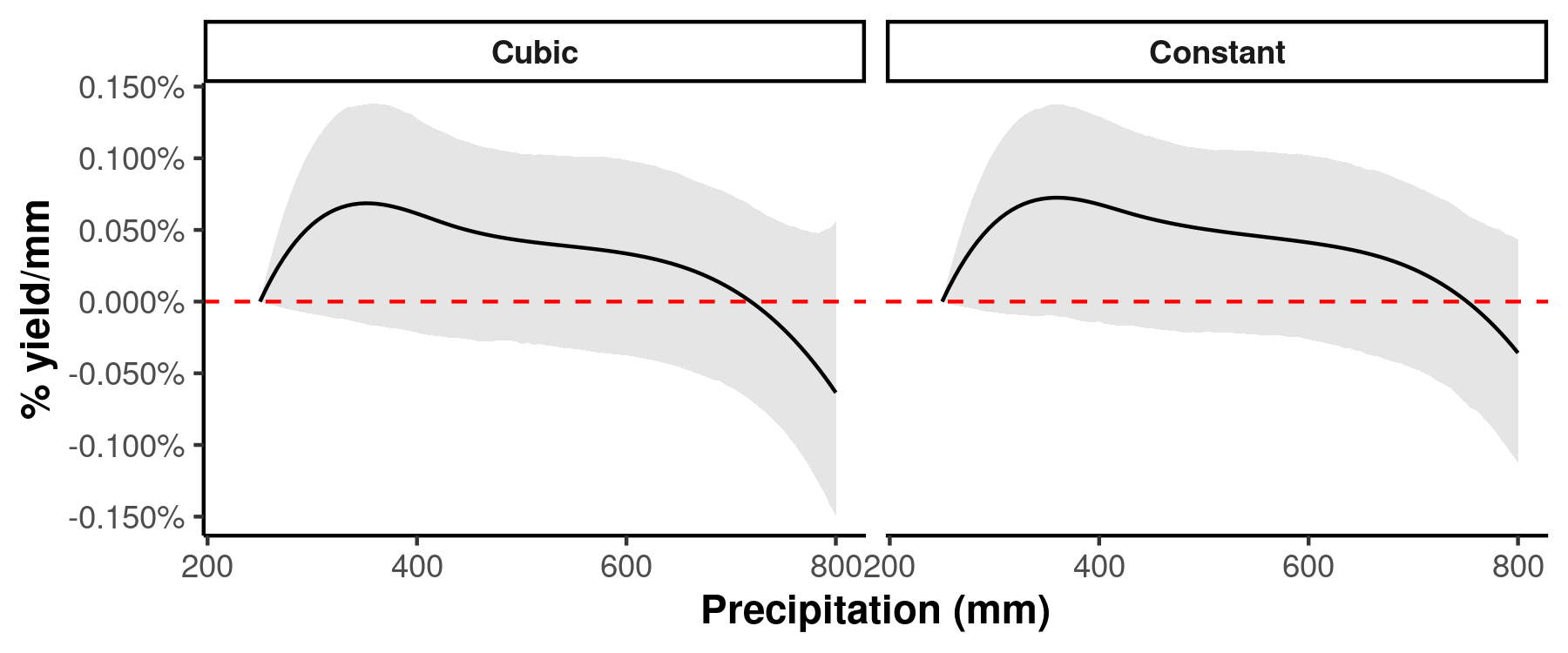

Supplement: S8 Fig — Effects of precipitation on percentage yield for models with differing representations of temperature effects. Solid lines indicate the mean and shaded areas 95% confidence bands for 2,000 block bootstraps. (PNG) [file pgen.1010799.s008.png]

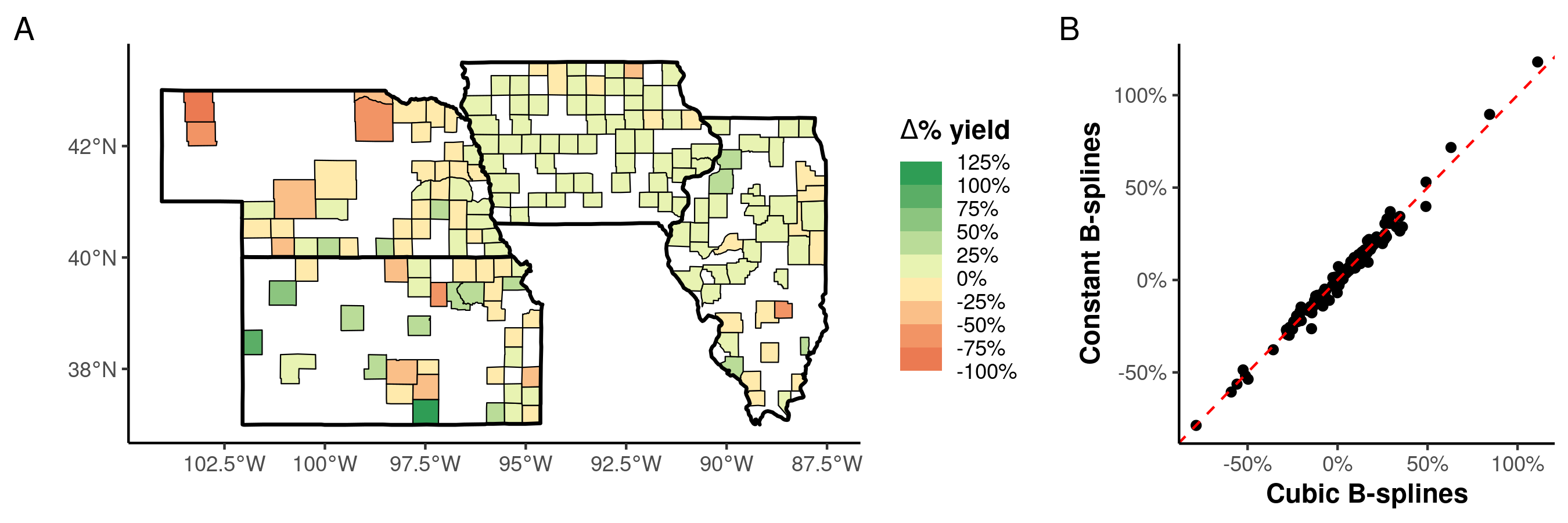

Supplement: S9 Fig — (A) Mean percentage effect of each county from the cubic B-spline model. The map was generated using the ‘maps’ R package (https://cran.r-project.org/package=maps). (B) Comparison of mean location effects in models with differing representations of temperature effects. The dashed red line indicates equality. In both panels, the means of 2,000 block bootstraps are shown. (PNG) [file pgen.1010799.s009.png]

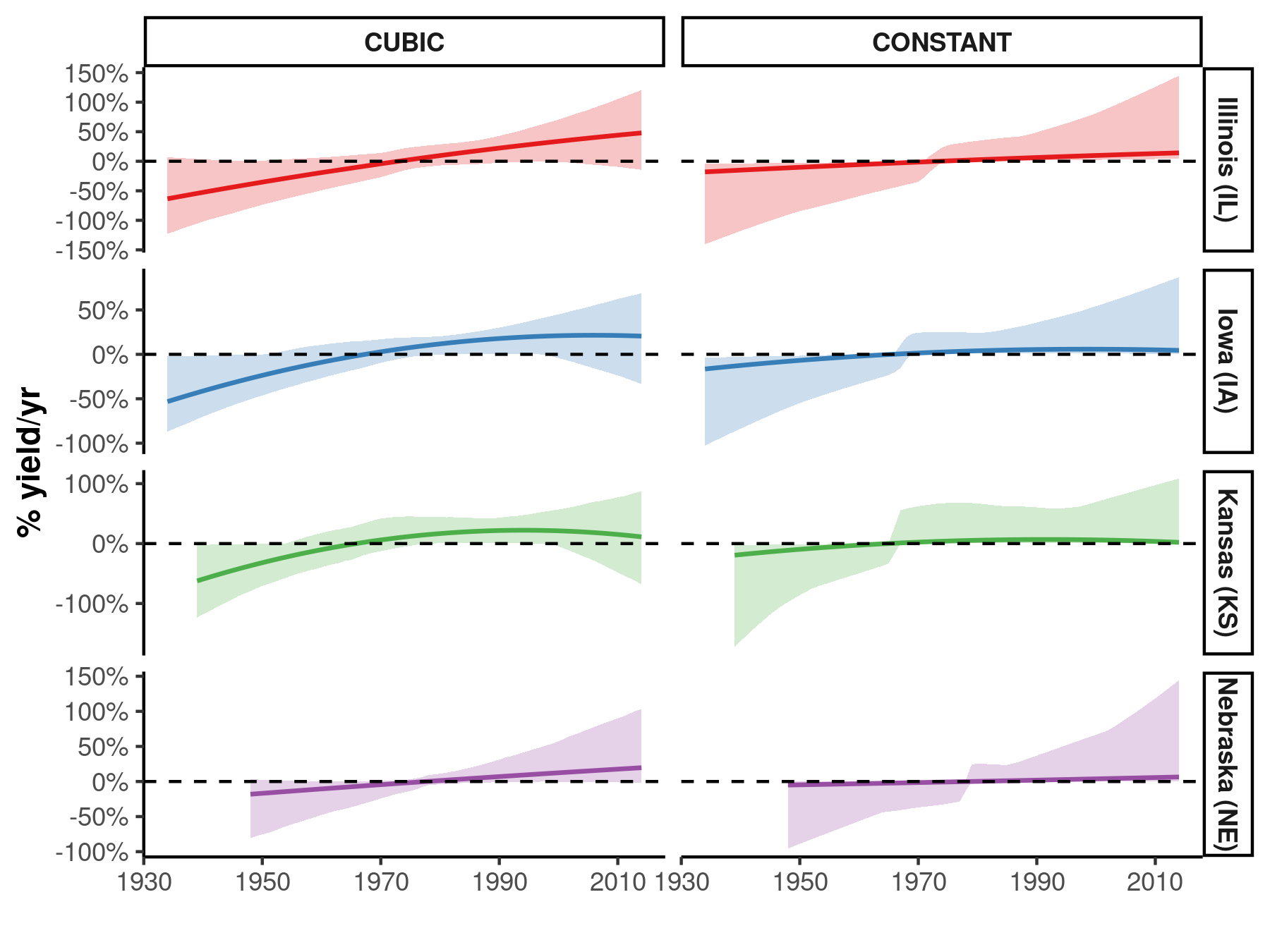

Supplement: S10 Fig — Each panel depicts the effect of year on percentage yield for the indicated state relative to 1981, the mean trial year in the dataset. Effects were estimated as the random regression of natural log-transformed yield (bu/a) on an orthogonal, quadratic polynomial of trial year. Solid lines indicate the mean and shaded regions the 95% confidence bands of 2,000 block bootstraps. (PNG) [file pgen.1010799.s010.png]

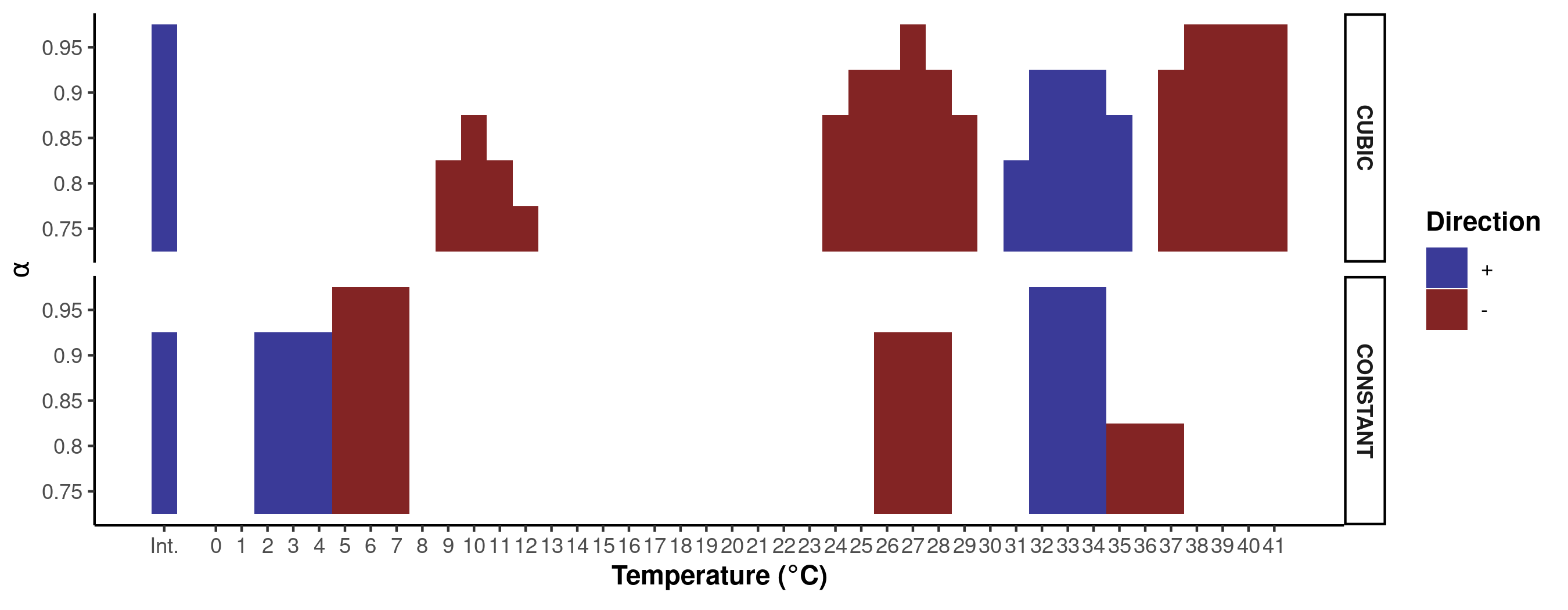

Supplement: S11 Fig — Results shown in this figure are for exposure distributions and response functions parameterized as constant B-splines (see “Methods” and Fig 2 for an alternative parameterization). Point estimates and confidence intervals/bands are based on 2,000 block bootstraps. (A) Population mean temperature response function for 4,730 maize hybrids. The solid line indicates the mean fixed effect coefficient function, and confidence bands at two confidence levels are shown. The vertical difference between any two points on the function indicates the percentage change in yield associated with substituting one-hour of exposure within one 3°C temperature bin for another. (B) The left panel indicates the mean (dot) and 90% (thick line) and 95% (thin line) confidence intervals for selection on the random hybrid intercepts. The right panel illustrates the selection function on breeding values for the temperature response functions. Figure elements have the same meaning as in panel (A). (C) Mean correlation between random hybrid intercepts (li) and temperature response function coefficients (βih) for hybrids grouped by year of introduction. Labels indicate significance at the 90% (^) or 95% (*) confidence levels. (D) Centered and scaled time series of weighted mean hybrid cohort temperature response function coefficients. Each curve represents the time series for response to a 3°C temperature bin smoothed by a cubic B-spline with seven internal knots. (PNG) [file pgen.1010799.s011.png]

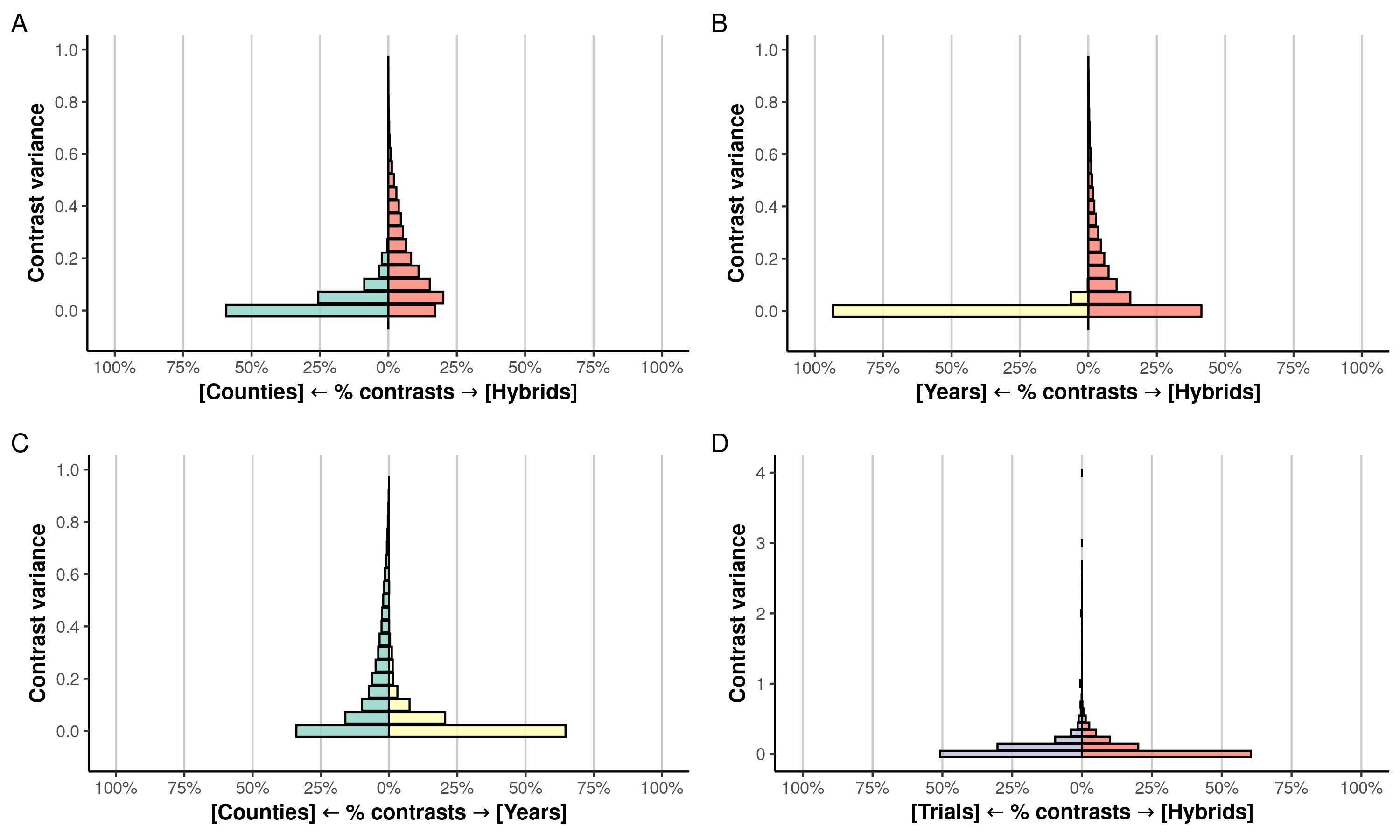

Supplement: S12 Fig — Each panel shows the distribution of predicted contrast variances for the difference between two levels of a factor when distributed over the levels of the other indicated factor. For example, the left side of panel A shows the contrast variances of counties when distributed over hybrids. The bin width is 0.05 in panels A-C and 0.1 in panel D. There are 172 levels of county; 81 levels of year; 4,730 levels of hybrid; and 2,581 levels of trial. See “Methods” for more details. (PNG) [file pgen.1010799.s012.png]

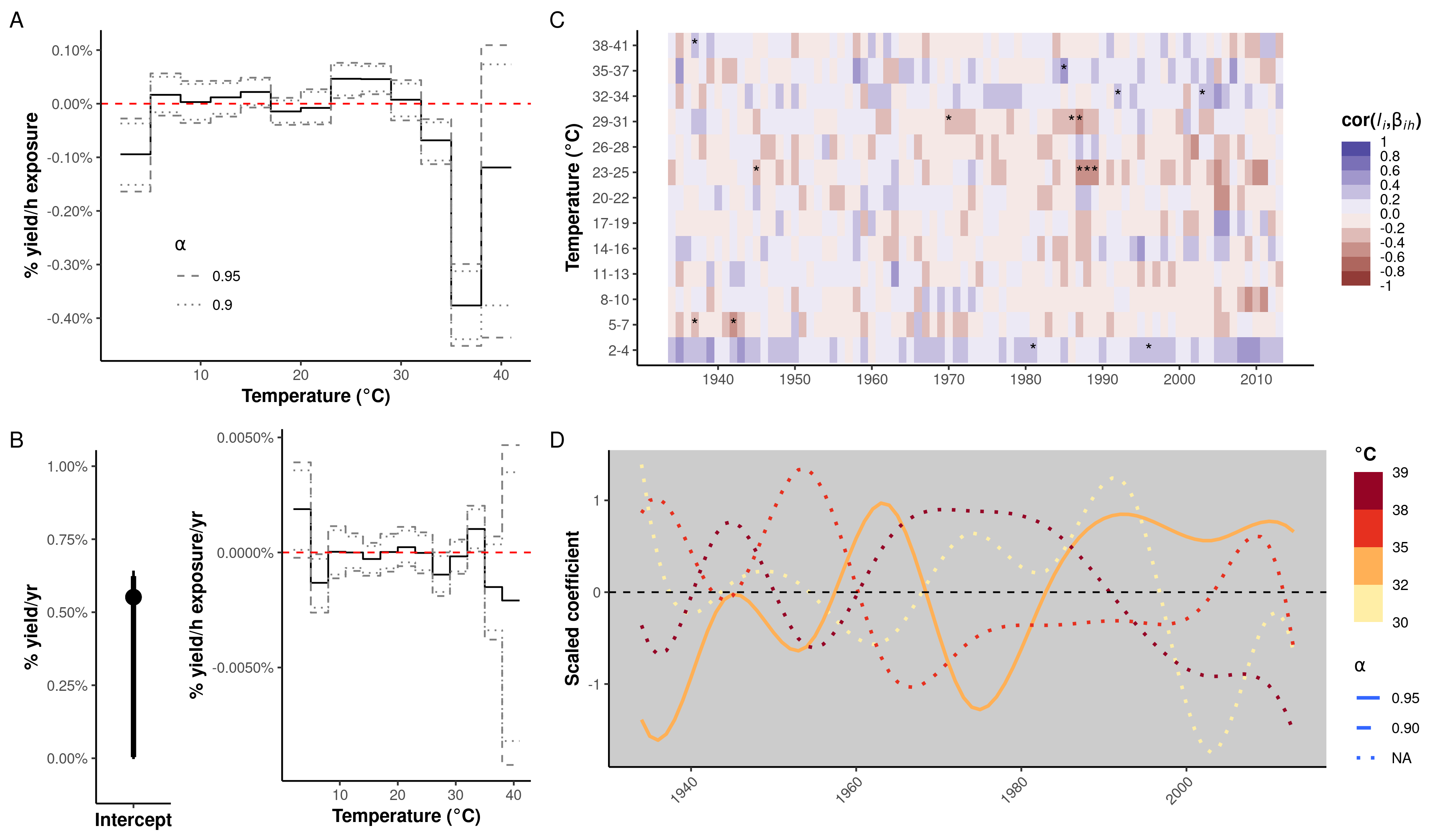

Supplement: S13 Fig — For the estimation of directional selection, see “Methods”. The direction is the sign of the mean slope of 2,000 block bootstraps. Significance is defined as the exclusion of zero by the (100 × α)% confidence interval of 2,000 block bootstraps as indicated on the y-axis. (PNG) [file pgen.1010799.s013.png]

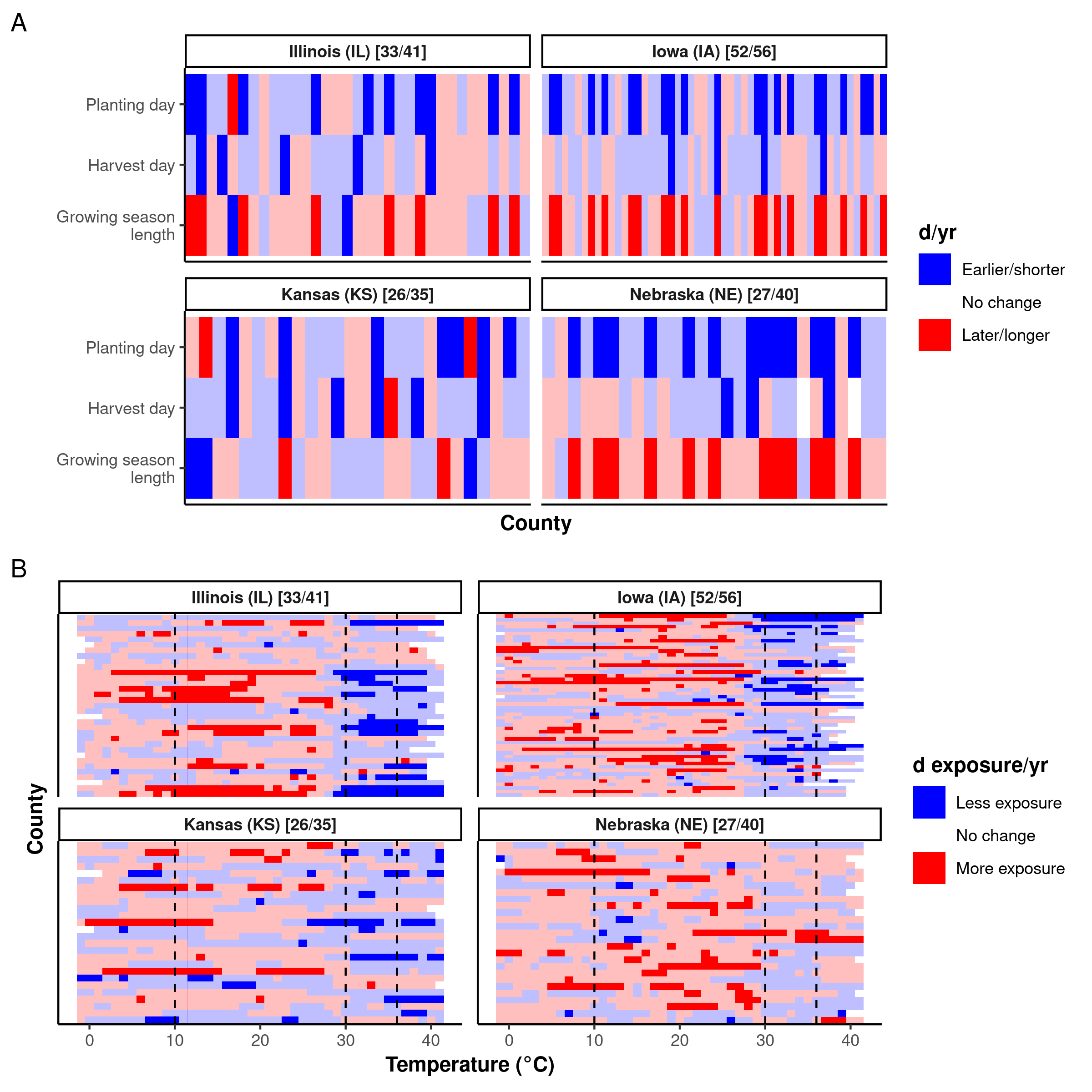

Supplement: S14 Fig — (A) Trends in Julian planting and harvest dates and growing season length by county. Only counties with at least four years of data are shown. Color indicates the sign of the slope of a linear regression of the indicated variable on year. Solid tiles indicate significance at an uncorrected p < 0.05 level. (B) Trends in temperature exposure time (in days) by county. Figure elements are the same as in panel A. Vertical dashed lines indicate the optimal temperature bounds for growing degree day calculations (10 and 30°C) and the moderate/severe heat stress breakpoint (36°C) identified in the main text. (PNG) [file pgen.1010799.s014.png]

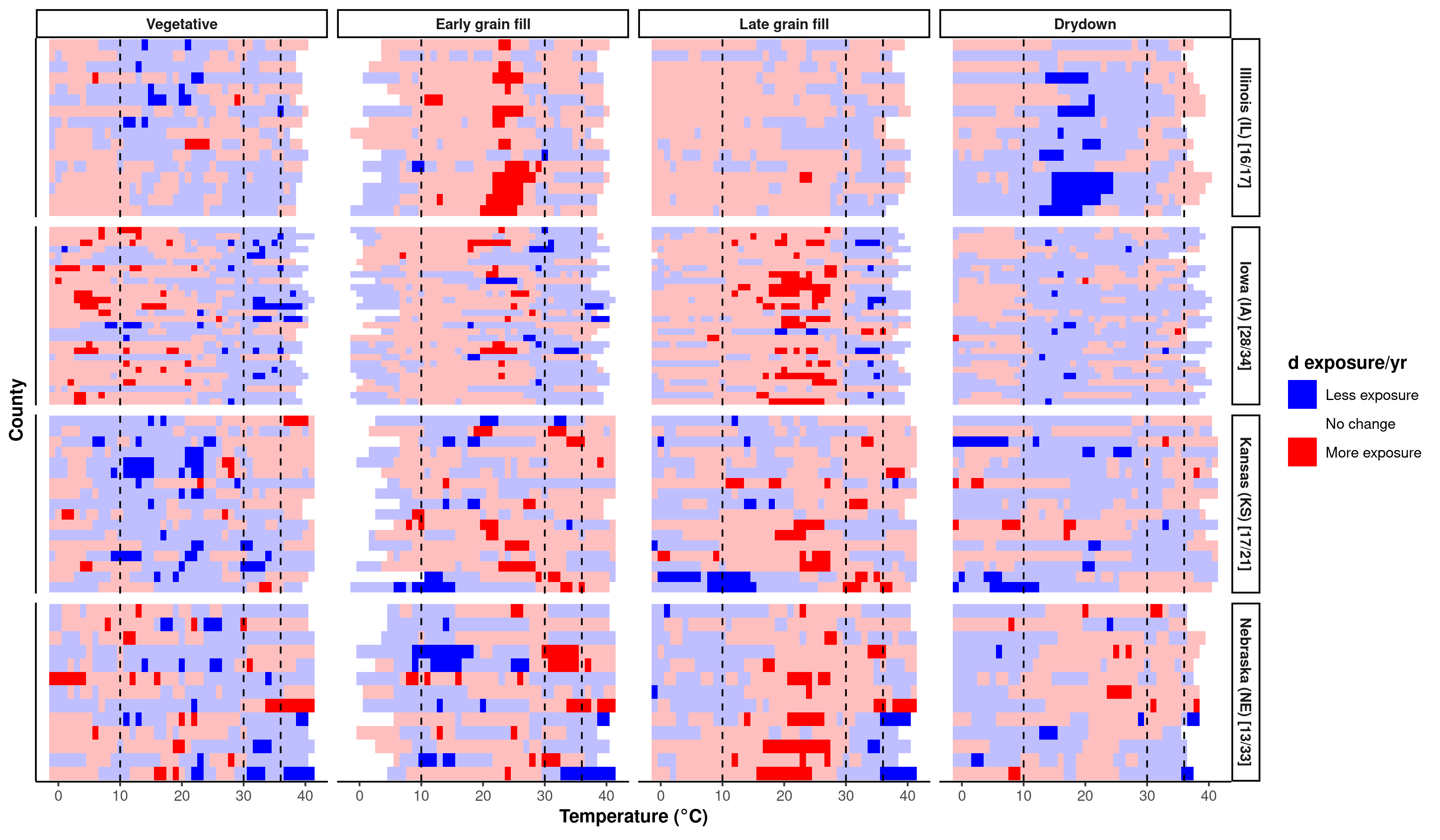

Supplement: S15 Fig — Only counties with at least four years of data are shown. Color indicates the sign of the slope of a linear regression of the indicated variable on year. Solid tiles indicate significance at an uncorrected p < 0.05 level. Vertical dashed lines indicate the optimal temperature bounds for growing degree day calculations (10 and 30°C) and the moderate/severe heat stress breakpoint (36°C) identified in the main text. Stages are defined as: “Vegetative” = planting to silking; “Early grain fill” = silking to dough; “Late grain fill” = dough to maturity; “Drydown” = maturity to harvest. (PNG) [file pgen.1010799.s015.png]

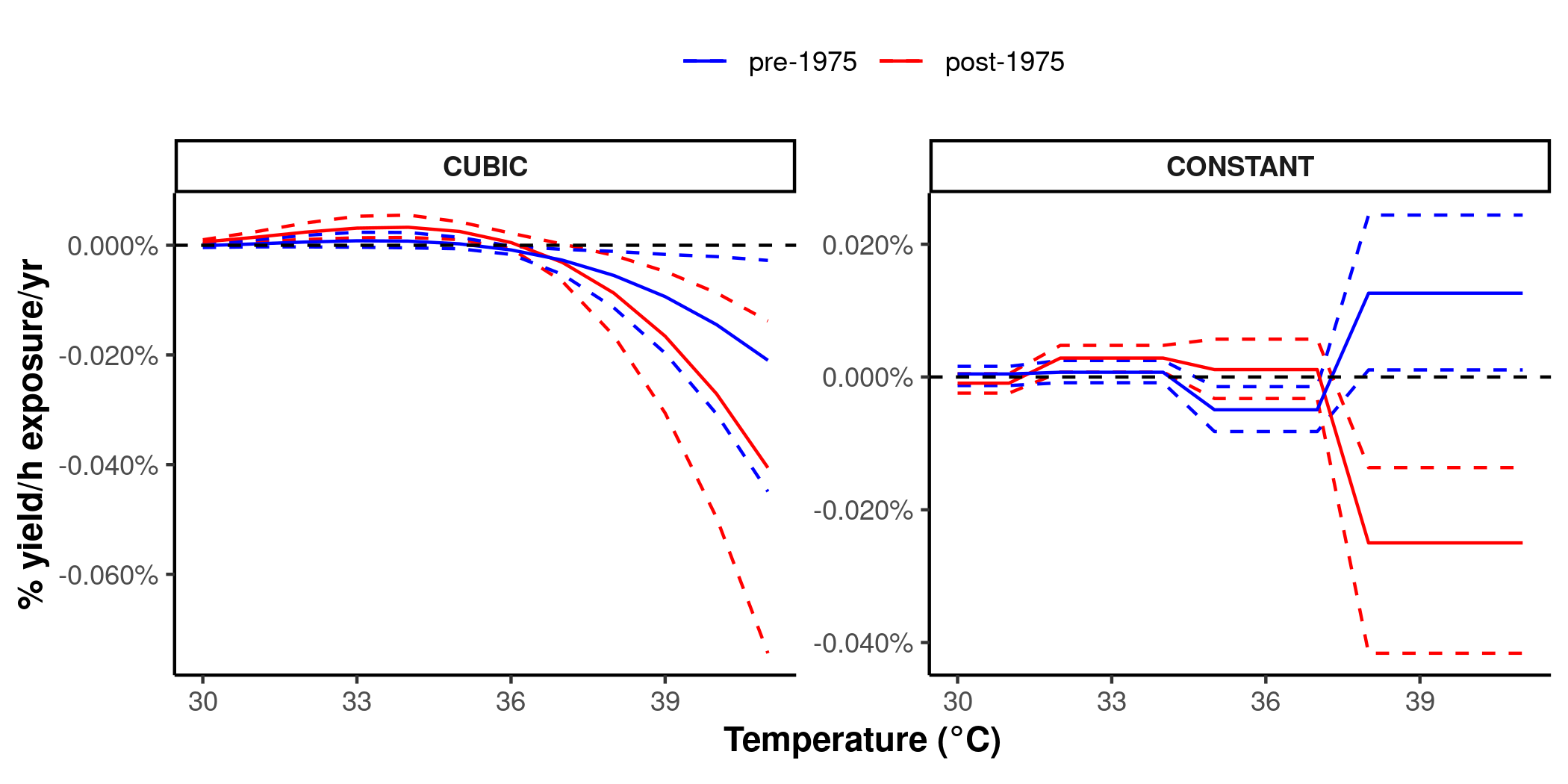

Supplement: S16 Fig — The strength (cubic model) and direction (constant model) of selection differ between hybrids introduced before and after 1975. Results for the cubic B-spline parameterization are consistent in direction with those given in Fig 2B (right panel). Results for the constant B-spline parameterization show changes in direction for the different time periods and increased statistical significance compared with those given in S8B Fig (right panel). Solid lines indicate the mean strength and direction of selection, and dashed lines indicate the 95% confidence bands. Point estimates and confidence bands are based on 2,000 block bootstraps. (PNG) [file pgen.1010799.s016.png]

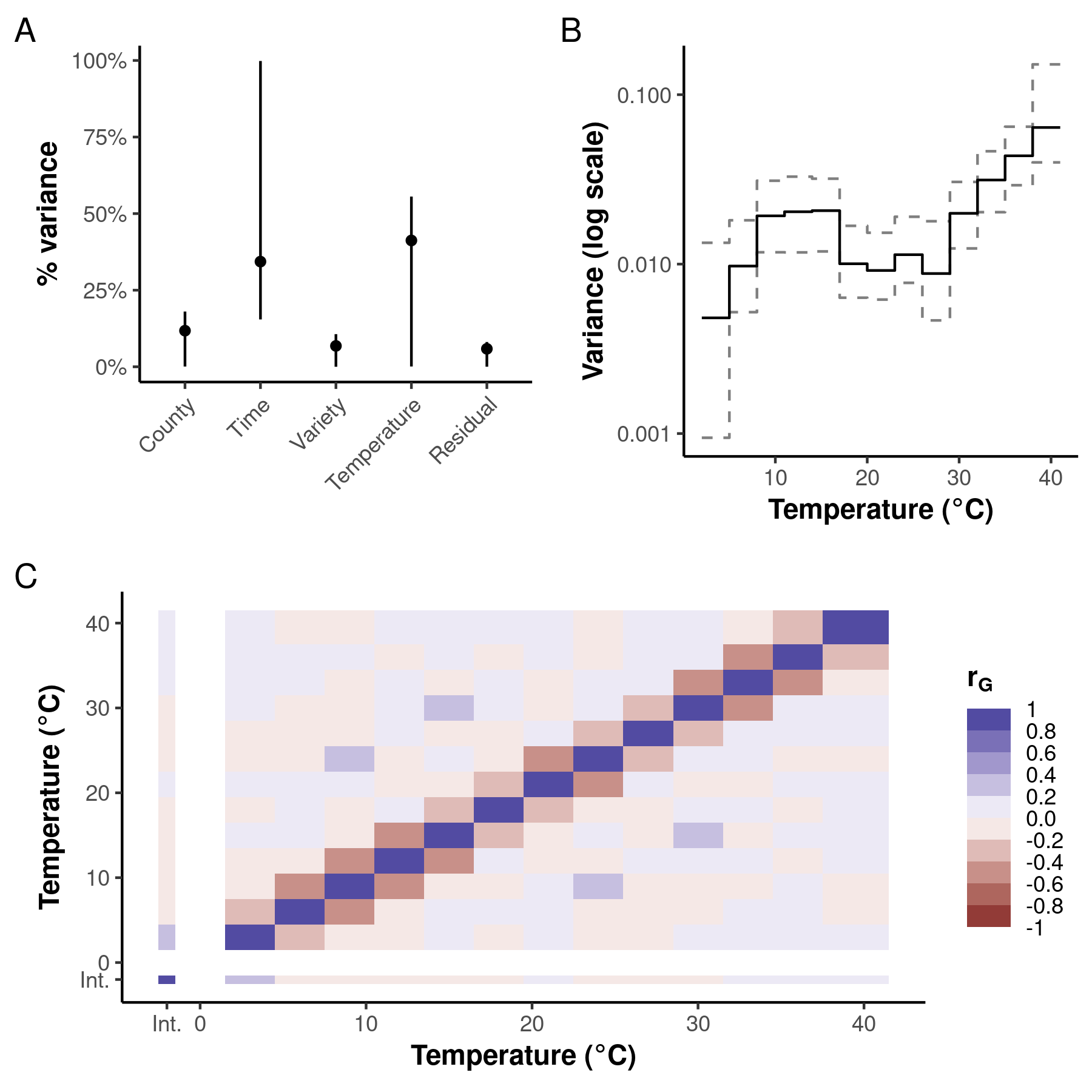

Supplement: S17 Fig — Results shown are based on 2,000 block bootstraps for the model where exposure distributions and response functions are parameterized by constant B-splines (see “Methods” and Fig 3 for an alternative parameterization). (A) Percentage variance attributed to random effects. “Variety” indicates the genetic variance in hybrid intercepts; “Temperature,” the combined genetic variance for responses to all temperatures. Points indicate means and lines the 95% confidence intervals. (B) Genetic variance of breeding values for temperature response functions (N.B. the logarithmic scale). The solid line indicates the mean; dashed lines, the 95% confidence bands. (C) Genetic correlation function for the correlation between breeding values for temperature responses at different temperatures and the hybrid intercepts (bottom-most and leftmost row and column, respectively). The bootstrapped mean function is depicted. (PNG) [file pgen.1010799.s017.png]

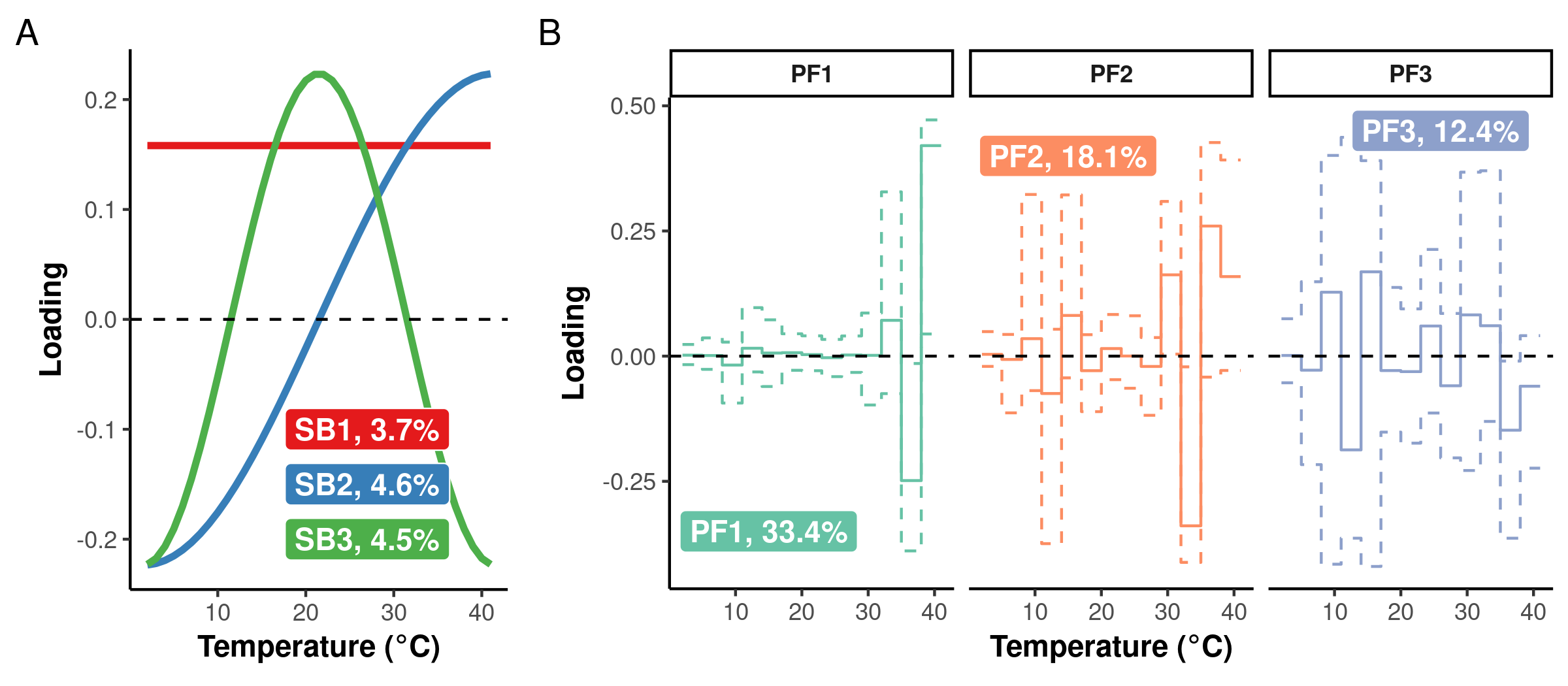

Supplement: S18 Fig — Results shown are based on 2,000 block bootstraps for the model where exposure distributions and response functions are parameterized by constant B-splines (see “Methods” and Fig 4 for an alternative parameterization). (A) Loadings for the first three simple basis (SB) functions of the genetic covariance function for temperature responses with the genetic variance explained by each function. The form of each SB function is determined by the choice of a simplicity metric in contrast to principal function analysis (PFA), which identifies functional responses of greatest variance. Each SB function describes genetic variation in a biological interesting direction: SB1 quantifies overall performance (i.e., log-yield); SB2 quantifies the strength of a tradeoff between low and high temperatures; and SB3 quantifies a generalist-specialist tradeoff. The percentage of genetic variance associated with each SB function is the bootstrapped mean. (B) Loadings for the first three principal functions (PF) of the genetic covariance function. The first three PFs are shown, but the first nine PFs are required to account for at least 95% of the total genetic variance in temperature responses. Solid lines indicate the bootstrapped mean; dashed lines, the 95% confidence bands. The percentage of genetic variance associated with each PF is the bootstrapped mean. (PNG) [file pgen.1010799.s018.png]
